# Sixth Annual Mediclinic Middle East Research Conference

**DOI:** 10.1097/MD.0000000000033794

**Published:** 2023-10-20

**AGENDA**

Day 1, Thursday March 16, 2023

**PODIUM PRESENTATION ABSTRACTS**

**Table:** 

TIME	DESCRIPTION		SPEAKER	
08:30 – 09:00	Registration Mini Breakfast & Coffee			
09:00 - 09:05	Introduction		Dr. Zakaullah Khan Senior Corporate Medical Director Mediclinic Middle East	
09:05 - 09:10	Opening Remarks		Dr. Pietie Loubser Chief Clinical Officer Mediclinic Middle East	
09:10 - 09:20	Address by Guest of Honour		Mr. Hein VanEck Chief Executive Officer Mediclinic Middle East	
09:20	Introduction to the Keynote Speaker		Dr. Rakshinda Mujeeb Research Projects Manager Mediclinic Middle East	
09:20 - 10:00	KEYNOTE SPEAKER: Why breast cancer screening with mammography is effective?		Dr. Jean Seely Head of Breast Imaging & Professor of Radiology University of Ottawa, Canada	
10:00 – 10:20	Coffee Break			
Podium Presentations – 1st Session, Day 1 Moderator: Dr. Saeed Rafii, Consultant Medical Oncologist Mediclinic City Hospital, Dubai				
10:20 – 10:40	MRI-guided vacuum-assisted breast biopsies: Our experience as the first private healthcare facility setting up the service in UAE.			Dr. Alexandra Economacos Specialist Radiologist Mediclinic City Hospital, Dubai
10:40 – 11:00	Randomized trial of surveillance with abbreviated MRI in women with a personal history of breast cancer– impact on patient anxiety and cancer detection.			Dr. Tasneem Alhassan Consultant Breast Radiologist Mediclinic Parkview Hospital, Dubai Dr. Jean Seely Head of Breast Imaging & Professor of Radiology University of Ottawa, Canada
11:00 – 11:20	Contrast-enhanced Spectral Mammography: the MCIT experience.			Dr. Alexandra Economacos Specialist Radiologist Mediclinic City Hospital, Dubai
11:20 – 11:40	Patterns and Trends of Colorectal Cancer in the United Arab Emirates: A retrospective cohort study, 2012- 2021.			Mr. Ali Al Najjar Medical Student Mohammed Bin Rashid University Of Medicine and Health Sciences, Dubai Mr. Ahmad Amrou Medical Student Mohammed Bin Rashid University Of Medicine and Health Sciences, Dubai
11:40 – 11:50	Q&A			
11:50 – 12:50	Lunch			
Podium Presentations – 2nd Session, Day 1 Moderator: Dr. Iram Siddiqui, Consultant Obstetrician & Gynaecologist, Fetal Medicine Mediclinic Al Noor Hospital				
12:50 – 13:10	Prediction of Covid-19 Patients’ Disposition and Prognosis Using National Early Warning Scores in A Tertiary Care Hospital, 2021: A Retrospective Case Series.	Ms. Asmaa Haj Husin Medical Student Mohammed Bin Rashid University Of Medicine and Health Sciences, Dubai		
13:10 – 13:30	Global and regional prevalence of multimorbidity in the adult population in community settings: A systematic review and meta-analysis.	Dr. Ahmed Hossain Professor University Of Sharjah		
13:30 – 13:50	Prevalence and associated risk factors of glaucoma among adults in Dubai, the United Arab Emirates – a retrospective study.	Ms. Maryam Jafari Medical Student Mohammed Bin Rashid University Of Medicine and Health Sciences, Dubai		
13:50 – 14:10	The Role of Inflammation and Oxidative Stress in the Management of Prediabetes.	Ms. Hibba Yousef Teaching Assistant Khalifa University, Abu Dhabi		
14:10 – 14:30	Initial experience of robotic ventral hernia repair at a single center.	Dr. Muhammad Umar Hospitalist Mediclinic City Hospital, Dubai		
14:30 – 14:40	Q&A			
14:40 – 16:30	WORKSHOP: Clinical Trials of the Future 2023 – 2053 • Introduction to study design (14:40 – 15:00) • Workshop on design of trials of future (15:00 – 15:15) • Discussion (15:15 – 16:30)	Dr. Marie Ibrahim Associate Director of Regulatory Affairs & Pharmacovigilance Director Insights Research Organization & Solutions G42, Abu Dhabi Ms. Nargis Azizali Kings college London, Dubai		

Day 2, Friday March 17, 2023

**Table:** 

TIME	DESCRIPTION	SPEAKER
09:40 – 10:00	The Use of Artificial Intelligence in Colonoscopy Improves Adenoma Detection Rates and inversely reduces the risk of interval colorectal cancer; First Comparative Study in UAE.	Dr. Mazin Al Jabiri Consultant Gastroenterologist Mediclinic Parkview Hospital, Dubai
10:00 – 10:20	Minithoracotomy versus Sternotomy in mitral valve surgery. Meta-analysis from recent matched and randomized studies.	Dr. Olivier Jegaden Consultant Cardiologist Mediclinic Parkview Hospital, Dubai
10:20 – 10:40	xDeepPolar: A new clinical tool for multi-parameter data integration to assess left ventricular ejection fraction.	Mr. Mohanad Alkhodari Research Associate Khalifa University, Abu Dhabi
10:40 – 10:50	Q&A	
Podium Presentations – 1st Session, Day 2 Moderator: Dr. Tabassum Parveen, Consultant Obstetrician & Gynaecologist Mediclinic Airport Road Hospital, Abu Dhabi Podium Presentations – 2nd Session, Day 2 Moderator: Dr. Rana M. Abou Mrad, Consultant Nephrologist Mediclinic Airport Road Hospital, Abu Dhabi		
10:50 – 11:10	The association between sleeping behavior, obesity, psychological depression, and eating habits among adolescents in the Emirate of Abu Dhabi – United Arab Emirates.	Dr. Rania Al Dweik Assistant Professor Abu Dhabi University
11:10 – 11:30	Correlation Between First Trimester Serum Uric Acid And Subsequent Development of Gestational Diabetes Mellitus.	Dr. Maha Majeed Medical Director Mediclinic Jowhara Hospital, Al Ain
11:30 – 11:50	The effect of beta-blocker therapy on heart rate variability for heart failure with preserved ejection fraction.	Ms. Shiza Saleem Research Assistant Khalifa University, Abu Dhabi
11:50 – 12:00	Q&A	
Prize Distribution Moderator: Ms. Zineb Katache, Research Assistant Mediclinic Middle East, Dubai		
12:00 – 12:20	**Poster Presentation Award** Winner 1st runner up 2nd runner up **Dr. Edwin Hertzog’s Award** Best Researcher for the year 2023	Dr. Pietie Loubser Chief Clinical Officer Mediclinic Middle East
12:20 – 12:30	Closing Remarks	Dr. Pietie Loubser Chief Clinical Officer Mediclinic Middle East

**Figure:**
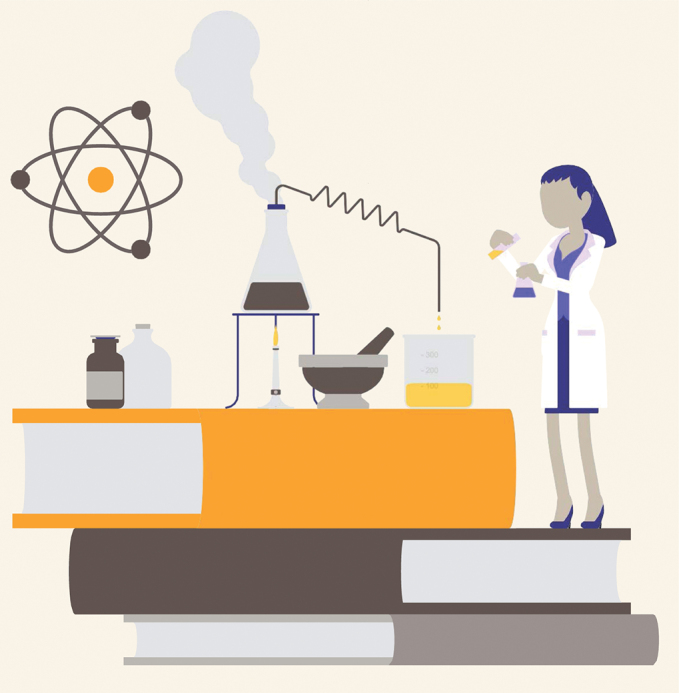


## MRI-GUIDED VACUUM-ASSISTED BREAST BIOPSIES: OUR EXPERIENCE AS THE FIRST PRIVATE HEALTHCARE FACILITY SETTING UP THE SERVICE IN UAE

**Figure:**
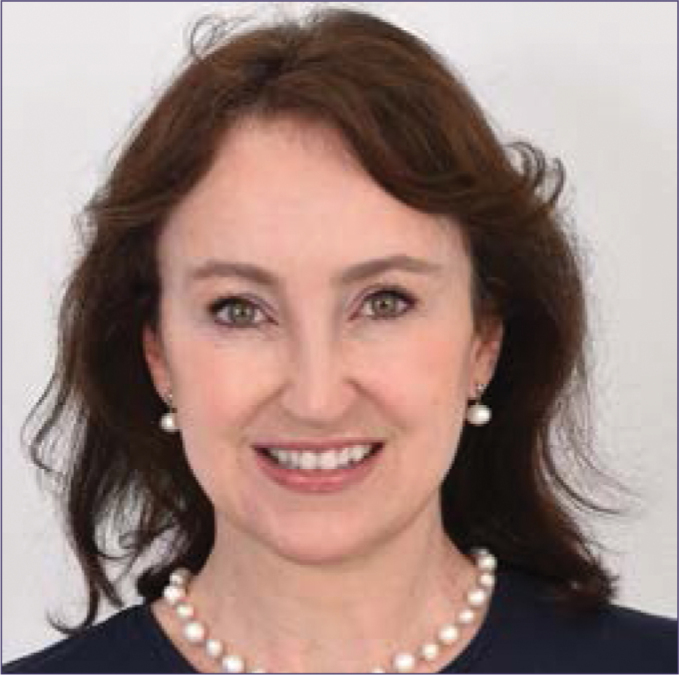


Dr. Alexandra Economacos

*Consultant Breast Radiologist, Mediclinic City Hospital, Dubai, UAE*.

Dr. Leila Ismail

Dr. Konstantia Diana Stavrou Dr. Yee Ting Sim

*Consultant Breast Radiologist, Mediclinic City Hospital, Dubai, UAE.*

**Objective:** MRI guided vacuum biopsy-VAB-is used to diagnose the causes of indeterminate enhancing lesions identified on contrast breast MRI that cannot be further assessed under mammography or ultrasound. Our department is the first private facility to offer this option in the UAE and we wished to determine the safety and efficacy of this service for patient disease management)

**Methods:** Retrospective audit of all MRI-guided breast VAB’s in our hospital from February 2020 to September 2022 was conducted. Data collated from Radiology Information System, biopsy and surgical pathology reports from Electronic Medical Records. Excluded from the audit are patients in whom the planning MRI sequences conducted on the day of VAB no longer show abnormal enhancement.

**Results:** Total of 50 MRI-guided VAB’s were performed since inception of the service. Technical success rate is 100%. The histopathology outcomes are: benign, B2 (n = 35, 70%), lesions of uncertain malignant potential, B3 (n=9, 18%), malignant, B5 (n=5, 10%). To date, there are no major peri- or post-procedural complications requiring surgical intervention. Minor complications include haematoma (although the haematoma rate is not higher than VAB’s performed under stereotactic or ultrasound guidance), minor skin tear requiring suture.

**Conclusion:** MRI VAB is a safe and accurate method to obtain histological diagnosis of indeterminate enhancing breast lesions at our facility with limited complications. With this additional service, unnecessary extensive breast surgery has been avoided, with good patient outcomes.

## THE USE OF ARTIFICIAL INTELLIGENCE IN COLONOSCOPY IMPROVES ADENOMA DETECTION RATES AND INVERSELY REDUCES THE RISK OF INTERVAL COLORECTAL CANCER; FIRST COMPARATIVE STUDY IN UAE;415

**Figure:**
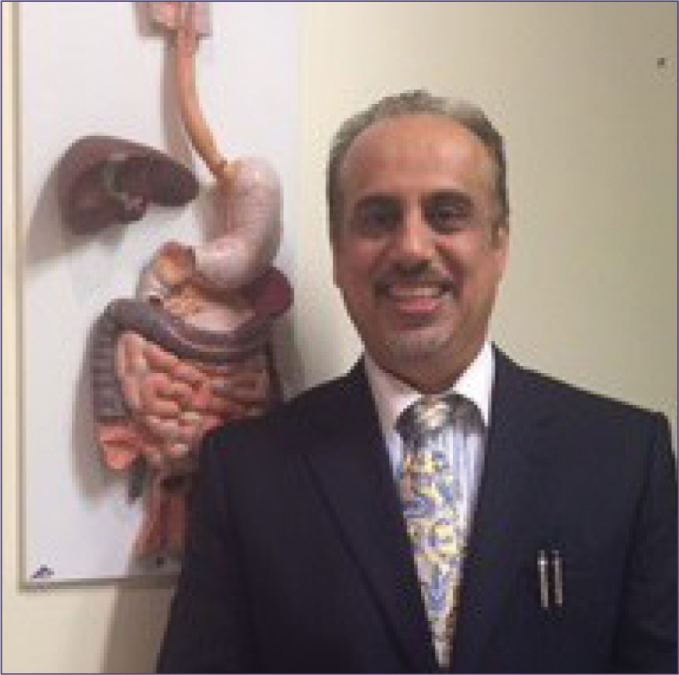


Dr. Mazin Aljabiri

*Consultant Gastroenterologist*

*Mediclinic Parkview Hospital United Arab Emirates-Dubai*

*Usama Warshow Alexandra Deduchova, Rola Saadi, Ammar Al Hassan, Joyce Villanueva, Jam Tomagan, Archebal Alimagno, Liji George, Saranya Das, Joven Dadang, Kamille Paz, Allysa Badong, Antonette Santos.*

**Background:** Globally, colorectal cancer (CRC) is the third most commonly diagnosed cancer in males and the second in females, with the second-highest cancer mortality rate(1). Adenomas are a major precursor lesions for CRC and as such adenoma detection rate (ADR) is an important and well recognized quality indicator of colonoscopy-based screening & surveillance worldwide. ADR is defined as the fraction of patients undergoing first-time screening colonoscopy who have one or more conventional adenomas detected and identified by pathology. The recognised minimum quality standard in a mixed-gender screening population is 25%. It is estimated that for every 1% increase in ADR, a patient’s risk of developing colon cancer over the next year decreases by 3%(2). Therefore, recent evidence suggestions have aspired to ADR threshold up to 39-50% to provide increased protection against post-colonoscopy interval cancer occurrence(3,4,5) Furthermore, raising the ADR leads also to reduction in Adenoma Miss Rate (AMR) (6), Artificial intelligence-assisted colonoscopy (AIAC) has gained attention as a tool to assist with polyp detection during colonoscopy(6). Artificial intelligence-assisted colonoscopy (AIAC) systems are intended to address the issue of missed polyps during colonoscopy. The effect of AIAC on ADR during screening and surveillance colonoscopy has not previously been studied in United Arab Emirates (UAE).

**Aim:** To assess and compare, for the first time in UAE, the use AIAC and its effect ADR in patients undergoing screening or surveillance colonoscopy.

**Method:** A single-centre study at Mediclinic Parkview Hospital, Dubai, UAE. The AIAC system module was utilized by five experienced endoscopists. Outcomes of consecutive surveillance colonoscopies performed for the period Apr-Dec 2020 without the use of AI & Following introduction of AI for the period Jan-Sept 2022.Comparative analysis was carried out between the two cohorts, in particular ADR. Our centre ADR was consistently above 20% over the last 10 years

**Results:** Males 51%, females 49%.indications for colonoscopy were PR bleeding, Change in bowel habits, weight loss and surveillance for FHx of polyps/cancer or abnormal imaging. A total of AIAC prospectively were compared with 666 from retrospective cohort with AI unaided colonoscopies for the period between Apr –Dec 2020 & 858 AIAC procedures. The overall polyp detection rate (PDR) was r between groups (392 vs 640); the chi-square statistic is 7.7532. The p-value is 0.005362. Significant at p <.05.

The adenoma detection rate was significantly higher in the AIAC group compared to the and Unassisted colonoscopy is (31.25% vs 23.1%)

**Conclusion:** Our Study demonstrates that AIAC resulted in a statistically significant increase in ADR (8.15%) with Prior to AI ADR detection was 23% and Post introduction of AI in colonoscopy increase to 31.25%, demonstrating the value of AIAC in a real-life cohort as ADR is an established performance indicator in colonoscopy and are inversely associated with the risks of interval colorectal cancer.(7). We recommend that all public and private sector hospitals consider implementing AIAC to improve quality of colonoscopy and optimise ADR.

## PATTERNS AND TRENDS OF COLORECTAL CANCER IN THE UNITED ARAB EMIRATES: A RETROSPECTIVE COHORT STUDY

**Figure:**
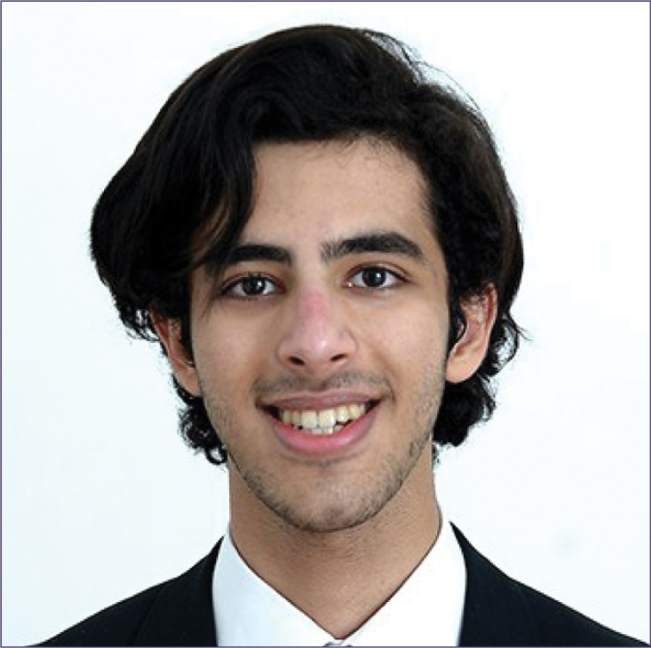


**Figure:**
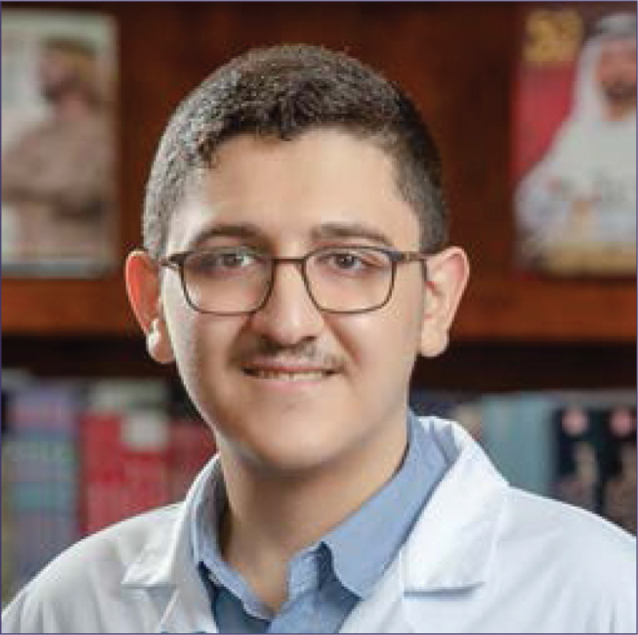


Ahmad Amrou & Ali Al-Najjar

*Medical Student, Mohammed Bin Rashid University Of Medicine and Health Sciences, Dubai*

Ali Al-Najjar, Rachid Kaddoura, Zainab AlAbdullah, Masa Alashkar,

*Neshteman Oghanna, Samuel Ho and Shroque Zaher (principal investigator)*

**ABSTRACT**

In 2020, colorectal cancer (CRC) was the third most diagnosed cancer and the second most fatal cancer worldwide. Data on 560 consecutive CRC cases from 2012- 2021 were analyzed and showed that most cases presented with symptoms at an advanced grade and stage, which indicates the need for increased CRC screening.

**Objective:** To assess patterns and trends of colorectal cancer (CRC) stratified by sociodemographic, histopathological, and clinical characteristics in a tertiary care hospital in Dubai.

**Methods:** Retrospective data from 560 patients with a diagnosis of CRC in a tertiary care hospital in Dubai (UAE) between 2012 and 2021 were analyzed. Variables studied included sociodemographic, histopathologic, and clinical.

**Results:** The frequency of CRC was highest among males compared to females, (56.4% vs 43.4%). The mean age of patients was 54.8 years old (SD = 13.7). Common nationalities included Emirati (21.6%), Indian (10.4%), and UK (8.8%). The predominant histological type of tumor was adenocarcinoma with 435/445 (97.8%) cases, followed by neuroendocrine tumor with 4/445 (0.9%), and mixed adeno-neuroendocrine and low grade appendiceal mucinous neoplasm, with 2/445 cases each (0.4%). At presentation 175/406 (43.1%) had locally advanced disease (pT4) and 134/424 (31.6%) had metastatic disease. The most common mode of clinical presentation was change in bowel habit in 160/384 (41.6%), followed by rectal bleeding in 156/384 (40.6%). Only 6.8% (26/384) presented with a positive fecal occult blood test through screening.

**Conclusion:** Most cases of CRC in this series are diagnosed at an advanced grade and stage. These data indicate the urgent need for increased CRC education and screening efforts.

## THE ROLE OF INFLAMMATION AND OXIDATIVE STRESS IN THE MANAGEMENT OF PREDIABETES

Hibba Yousef

*Teaching Assistant, Khalifa University, Abu Dhabi*

Ahsan H Khandoker, Herbert F Jelinek

*Department of Biomedical Engineering, Khalifa University, Abu Dhabi, United Arab Emirates*

Samuel F Feng

*Department of Science and Engineering, Sorbonne University Abu Dhabi, Abu Dhabi, United Arab Emirates*

**Background:** Primary prevention of type II diabetes mellitus is mainly based on lifestyle interventions, followed by pharmacological intervention with hypoglycemic agents in resistant cases. However, the success of lifestyle modifications may not translate as well from clinical trials to daily life, and hypoglycemic agents are often accompanied by side effects. Chronic inflammation and oxidative stress are largely recognized as the underlying mechanisms behind diabetes development, however, anti-inflammatory and antioxidant drugs have yet to approved for management of prediabetes.

**Objective:** To identify important oxidative stress and inflammatory biomarkers, alongside blood glucose levels, for quantification of the four-year and two-year risk of type II diabetes incidence. Such biomarkers can elucidate important mechanisms in the development of diabetes and provide insight into potential therapeutic targets.

**Methods:** Longitudinal analysis of 237 patients was carried out utilizing baseline levels of inflammatory and

oxidative stress biomarkers, along with body mass index and fasting blood glucose levels. Thirteen patients progressed to diabetes (within two years or four years), while 224 patients remained normoglycemic. Two logistic regression models were constructed with best subset selection to identify the most influential biomarkers.

**Results:** Both the two-year and four-year risk models revealed a stronger association between inflammatory and oxidative stress biomarkers with disease progression than blood glucose levels. Important biomarkers included interleukin-1b, oxidized glutathione, Humanin and 8-isoprostane, among others. Both the four-year and two-year models displayed high accuracy of 84% and 77%, respectively.

**Conclusion:** Given the strong association of the aforementioned biomarkers with diabetes incidence, it may be

prudent to extend preventive measures to include monitoring of inflammatory and oxidative stress levels of prediabetic patients, in addition to exploring therapies specifically targeting these processes

## PREDICTION OF COVID-19 PATIENTS’ DISPOSITION AND PROGNOSIS USING NATIONAL EARLY WARNING SCORES IN A TERTIARY CARE HOSPITAL, 2021: A RETROSPECTIVE CASE SERIES

**Figure:**
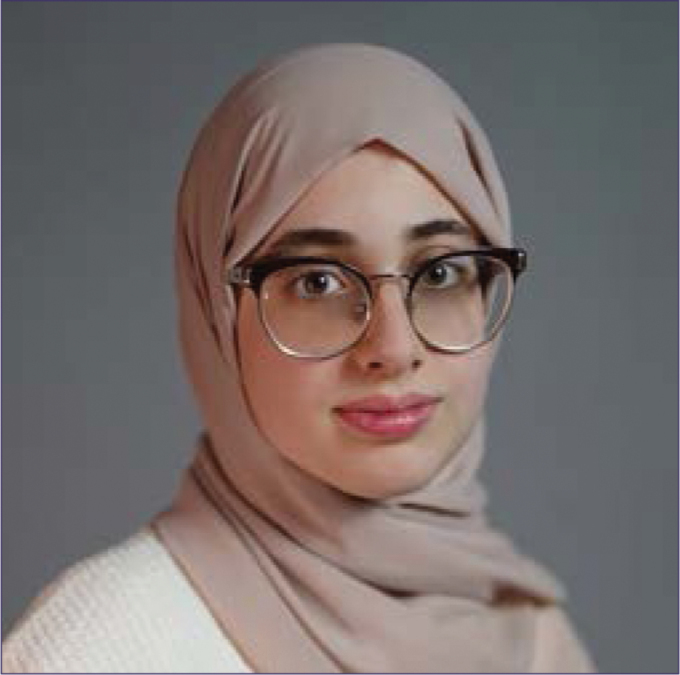


Asmaa Haj Husin

*3rd Year Medical Student, Mohammed Bin Rashid University of Medicine and Health Sciences,*

Hind Ahrari

*3rd Year Medical Student, Mohammed Bin Rashid University of Medicine and Health Sciences,*

Jeffrey Keep

*Head of Emergency Department, Mediclinic Parkview Hospital*

**Objective:** This study aimed to evaluate the performance of the National Early Warning Score 2 (NEWS-2) at emergency department (ED) triage to predict the admission or discharge of patients with suspected COVID-19.

**Methods:** Retrospective data from electronic health records of 300 COVID-19 positive patients who presented to Mediclinic Parkview Hospital ED between 1 January 2021 and 30 June 2021 were analyzed. Variables collected were age, gender, body mass index, vital signs, and disposition. Statistical analysis was used to estimate the ability of NEWS-2 to predict COVID-19 patients’ disposition.

**Results:** 300 consecutive patients who tested positive for COVID-19 by PCR testing were included. Statistical analysis was performed to calculate the sensitivities and specificities of each NEWS-2 score and plotted on a receiver-operator characteristic (ROC) curve. NEWS-2, with a cut-off value of 2, predicted hospital admission with 86% sensitivity and 75% specificity. It achieved an average area under the curve of 0.86 for predicting outcomes at 24 to 72 h from the time of initial presentation to the ED.

**Conclusions:** The NEWS-2 has high sensitivity and specificity to predict the disposition of patients with COVID-19. Our results support the use of NEWS-2 at ED triage as a valuable predictor of either admission or discharge from the ED.

## CONTRAST ENHANCED SPECTRAL MAMMOGRAPHY- THE MCIT EXPERIENCE

**Figure:**
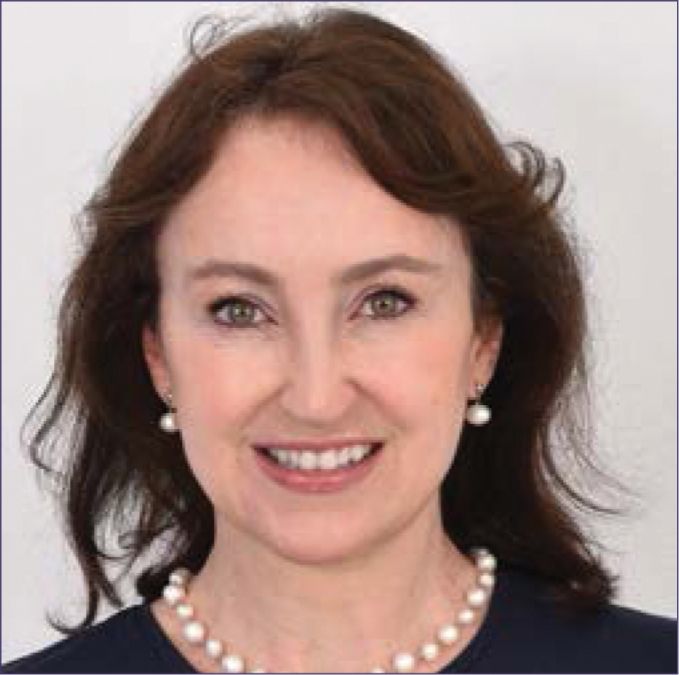


Dr. Alexandra Economacos

*Consultant Breast Radiologist, Mediclinic City Hospital, Dubai, UAE.*

Dr Leila Ismail

Dr Konstantia Diana Stavrou Dr. Yee Ting Sim

*Consultant Breast Radiologist, Mediclinic City Hospital, Dubai, UAE*

**Objective:** Contrast-enhanced spectral mammography (CESM) is emerging as a credible alternative to contras- enhanced breast MRI in some clinical settings. Dedicated equipment for this procedure at our facility has aided patients who are unable to undergo breast MRI to receive a fast, accessible imaging solution. We aim to provide a pictorial review of the benefits, pitfalls and imaging artefacts of CESM.

**Methods:** Retrospective review of all CESM conducted in our hospital from Sept 2020 to September 2022. Data collated from Radiology Information System and PACS. Representative cases were selected to highlight the most common indications and contraindications as well as illustrate artefact issues and imaging pitfalls encountered in our initial learning curve.

**Results:** To date, we have no patient complications arising from CESM conducted in our unit. We found CESM of superior diagnostic value to conventional mammography, particularly in patients with dense breasts. Most common CESM indication in our hospital is for problem solving and staging of breast cancer when MRI is contraindicated. The two main artefacts demonstrated are rim and ripple artefact. We found CESM of no diagnostic advantage in free silicone injected breasts

**Conclusions:** CESM has proven itself to be a fast and credible option at our facility for reviewing the breast in the settings of problem solving and cancer staging particularly in patients who are unable to undergo breast MRI. We would encourage a wider use of CESM as first-line breast imaging in younger symptomatic patients.

## PREVALENCE AND ASSOCIATED RISK FACTORS OF GLAUCOMA AMONG ADULTS IN DUBAI, THE UNITED ARAB EMIRATES – A RETROSPECTIVE STUDY

**Figure:**
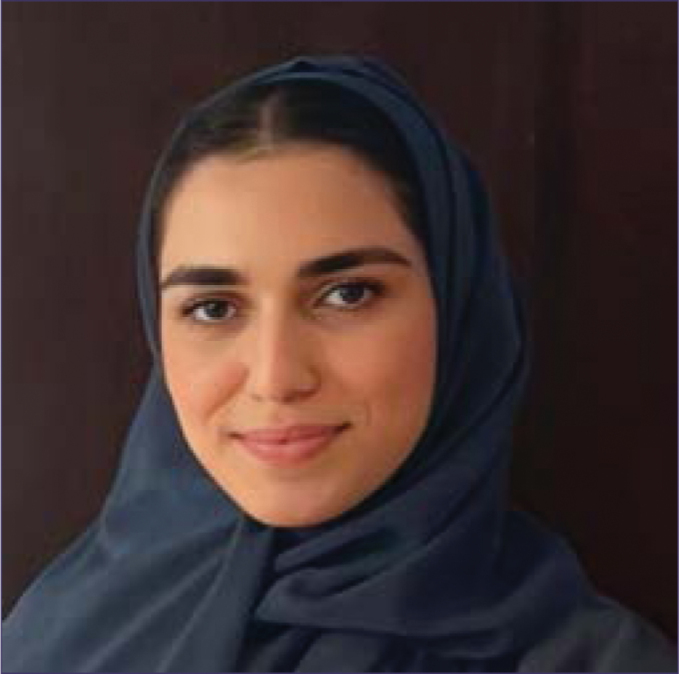


Maryam Jafari

*Mohammed Bin Rashid University of Medicine and Health Sciences*

Pramod T. Warhekar

Mediclinic City Hospital

Sarah Alkabbani

*Mohammed Bin Rashid University of Medicine and Health Sciences*

Sandeep Thakur

*Mediclinic City Hospital*

Jeyaseelan.Lakshmanan

*Mohammed Bin Rashid University of Medicine and Health Sciences*

**Objective:** Glaucoma is the second leading cause of blindness globally. Untreated glaucoma can permanently result in visual field defects, which can lead to blindness. Glaucoma can remain asymptomatic until late stages, and this results in high morbidity. There are no published studies on the epidemiology of glaucoma the United Arab Emirates. We aim to estimate the epidemiology of glaucoma in Dubai to guide future public health measures.

**Methods:** This is a hospital based retrospective study of all glaucoma patients aged 18 years and above that presented to outpatient department between 2017-2021. Variables include: prevalence of the glaucoma by type, intraocular pressure, visual acuity, visual fields, pachymetry, cup-to-disc ratio, and scanning laser ophthalmoscopy findings. Correlations were made between the variables.

**Results:** Of the 26,675 patients that visited ophthalmology outpatient department, 3265 patients were reviewed for glaucoma. After excluding pre-glaucoma patients and patients aged below 18 years, 554 (2.1%) were included in the study. The majority had primary open angle glaucoma (58.2%, 95%CI 54.1%-62.3%) and were mostly males (332, 60%) aged 53 ± 14 years (range, 20–98 years). Secondary glaucoma was present in 30% (164) of the patients. The mean intraocular pressure ranged from 6–55 mmHg. The glaucoma hemi field test was outside the normal limits in 80.8% of the patients. About 20% of the patients were diabetic, and 17.4% had hypertension.

**Conclusions:** Glaucoma is one of the leading causes of blindness worldwide. This is the first study to estimate the prevalence of glaucoma and its etiologies in a tertiary care hospital in Dubai. Some of the associated risk factors with glaucoma include age, diabetes, intra-ocular pressure.

## XDEEPPOLAR: A NEW CLINICAL TOOL FOR MULTI-PARAMETER DATA INTEGRATION TO ASSESS LEFT VENTRICULAR EJECTION FRACTION

**Figure:**
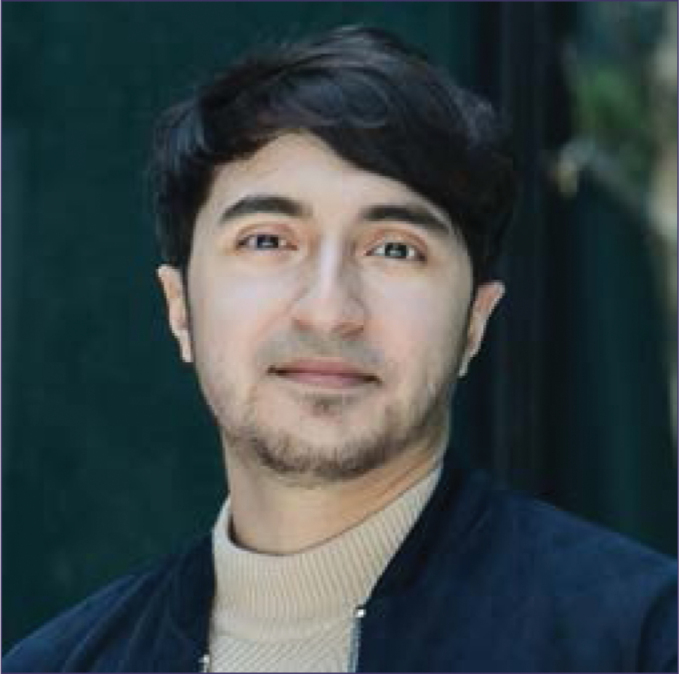


Mohanad Alkhodari

*Research Associate, Khalifa University, Abu Dhabi*

Ahsan H. Khandoker, Herbert F. Jelinek and Leontios J. Hadjileontiadis

**Background:** Heart failure (HF) is a multi-faceted and life-threatening syndrome that affects more than 64.3 million people worldwide. Gold standard clinical testing measures the left ventricular ejection fraction (LVEF) by echocardiography. Echocardiography results need to be interpreted by physicians in the presence of additional demographic/clinical data but, importantly, do not include changes associated with cardiac circadian rhythm.

**Objective:** xDeepPolar is a multi-parameter approach that combines patient data and 24-hour Holter recordings converted to hourly heart rate variability (HRV) feature as a single simplified source of information as a two-dimensional (2D), all-in- one color image for diagnostics and treatment planning.

**Methods:** The dataset included 303 patients from American and Greek medical centers. Patients with no congenital heart disease and with stable sinus rhythm were classified as preserved (>55%), mid-range (55%≥LVEF≥50%), and reduced (<50%) LVEF. A 24-hour Holter electrocardiography (ECG) and clinical history and demographics were included in the dataset.

**Results:** Using 7,575 color polar images in a trained deep learning model, xDeepPolar yielded mean sensitivity, specificity, and accuracy of 90.68%, 95.19%, and 92.62%, respectively. For pLVEF, the best classification was between the evening hours of 6-12 pm, while for rLVEF, afternoon hours (3-6 pm) had more impact on the decisions. mLVEF showed the best results at late-night and morning hours (1-7 am). Demographic information (age, sex, and BMI) were critical factors in predicting pLVEF and to a lesser extent, the mLVEF group. In contrast, these features were not crucial for identifying the rLVEF group.

**Conclusion:** xDeepPolar was merged into a user-friendly software application that translates into direct clinical use These findings potentiate xDeepPolar to serve as a powerful HF screening tool, including demographic/clinical data and circadian rhythm information that enhances single echocardiography tests towards individualized medicine.

## RANDOMIZED TRIAL OF SURVEILLANCE WITH ABBREVIATED MRI IN WOMEN WITH A PERSONAL HISTORY OF BREAST CANCER– IMPACT ON PATIENT ANXIETY AND CANCER DETECTION

**Figure:**
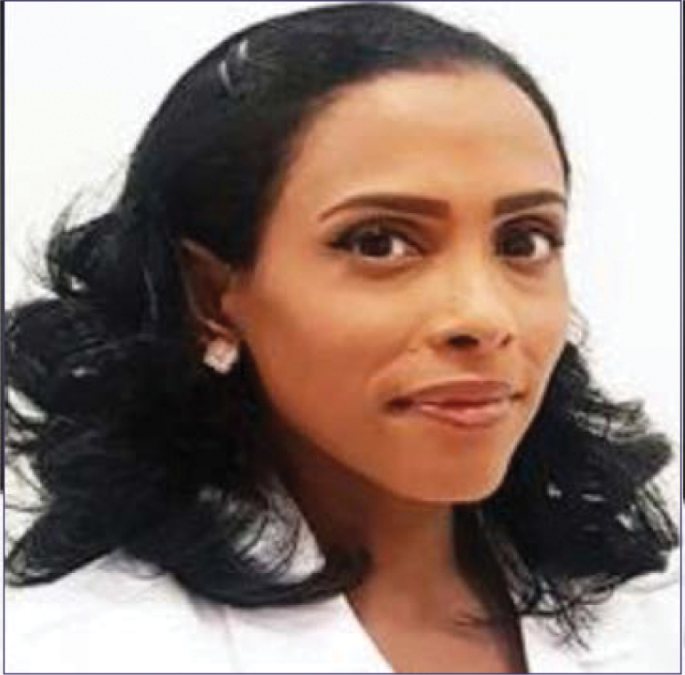


Dr. Tasneem Al Hassan

*Consultant Breast Radiologist, Mediclinic Parkview Hospital, Dubai*

**ABSTRACT**

This prospective controlled trial of parallel design was performed at a tertiary cancer center on asymptomatic women with a personal history of breast cancer (PHBC) who were randomized into two groups: routine surveillance with mammography (MG) or intervention of MG plus Abbreviated Breast MRI (A-MRI) in a 1:1 ratio. Primary outcome was anxiety measured by four validated questionnaires at three different time-points during the study. Other parameters including cancer detection rate (CDR) and positive predictive value for biopsy (PPV3) were compared between imaging modalities of MG and A-MRI.

198 patients were allocated to either MG alone (94) or MG plus A-MRI (104). No significant group difference merged for improvement in trait anxiety, worry and perceived health status.

A-MRI had higher incremental CDR (48/1000(5/104) vs MG 5/1000(1/198, p=0.01)) and higher biopsy rates (19.2% (20/104) vs MG 2.1% (2/94), p<0.00001) with no difference in PPV3 (A-MRI 28.6% (6/21) vs MG 16.7% (1/6,p>.05). There was no significant impact of A-MRI to patient anxiety or perceived health status. Compared to MG alone, A-MRI had significantly higher incremental cancer detection in PHBC. Despite a higher rate of biopsies, A-MRI had no demonstrable impact on anxiety, worry, and perceived health status.

## INITIAL EXPERIENCE OF ROBOTIC VENTRAL HERNIA REPAIR AT A SINGLE CENTER

**Figure:**
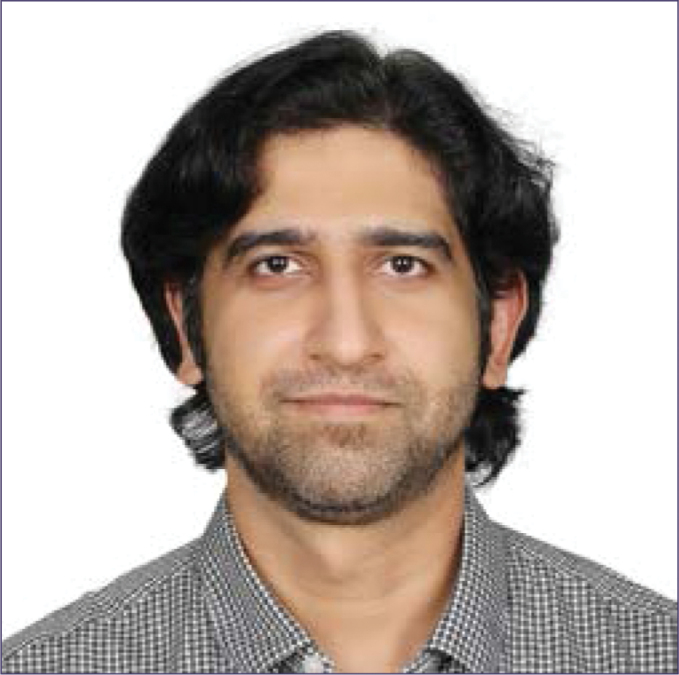


Dr. Muhammad Umar Younis

Dr Maliha Jaffar, Dr Faisel Ikram, Dr Hemant Vadeyar and Dr Roger Gerjy

*Department of General Surgery, Mediclinic City Hospital, Dubai, UAE*

**Objective:** Ventral hernia repair is a routine procedure in general surgery practice, the technique of which has evolved remarkably over the years. While many eponymous approaches were described and taught during residency years, Le Blanc et al set new standards when they described their laparoscopic ventral hernia repair and things have changed ever since1. Due to a number of advantages it offers, such as a shorter hospital stay, an earlier return to normal activities, comparable recurrence rates, cost effectiveness, and a notable decrease in wound-related problems, the laparoscopic technique has been widely embraced2,3. However, it can be ergonomically challenging in large hernial defects and do carry risks of intraabdominal adhesions and pain due to tacks. Robotic surgical systems have set to improve on these difficulties with surgeons now able to address complex hernias with satisfactory outcomes. We describe here our early experience of adoption of DaVinci Xi Robot for Ventral hernia repairs. While most of our repairs have been done using the single-dock robotic retromuscular ventral hernia repair (r-TARUP; robotic-TransAbdominal Retromuscular Umbilical Prosthesis)4, other complicated hernial defects have also required r-TAR (robotic transversus abdominus release) with optimal postoperative results.

**Methods:** A retrospective analysis of a single institution database was carried out and all robotic ventral hernia repairs from June 2020 to June 2022 were identified. The data parameters collected constituted patient demographics (age, gender, nationality), intraoperative variables (intraoperative time, docking and console times) and postoperative results (length of stay and early complications).

**Results:** Our initial review ascertained a total of 58 patients that underwent robotic repair of ventral hernia with a female predominance of 35 patients (57.89%) and a mean age of 50.84 years. Intraoperative times markedly improved as the robotic team got more familiar with the procedure. Docking times improved with a mean of 5.86 min to 3.35 min while the console times improved from a mean of 102.93 min to merely 84 min. Mean length of stay of patients was 1.389 days (Range 1-6). No complications were encountered and early results seemed promising on routine follow up.

**Conclusion**

Our initial experience upholds the superiority of Robotic repair of ventral hernia with excellent results. Although the retromuscular repair technique requires a learning curve and familiarity with the approach, the avoidance of sequelae of intraperitoneal mesh placement and postoperative pain with early return to activity are the rationale behind pursuing its implementation and perfection. Additionally, the robotic technique provides the surgeon with superior ergonomics, comfort and increased precision in undertaking suturing of the abdominal wall.

## GLOBAL AND REGIONAL PREVALENCE OF MULTIMORBIDITY IN THE ADULT POPULATION IN COMMUNITY SETTINGS: A SYSTEMATIC REVIEW AND META-ANALYSIS

**Figure:**
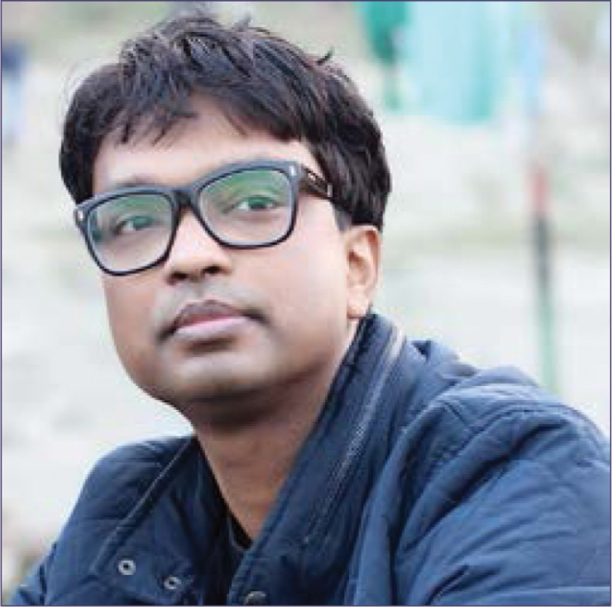


Dr Ahmed Hussain

*Professor, University of Sharjah*

**Background:** Knowing the prevalence of multimorbidity among adults across continents is a crucial piece of information for achieving Sustainable Development Goal 3.4, which calls for reducing premature death due to non-communicable diseases. A high prevalence of multimorbidity indicates high mortality and increased healthcare utilization. We aimed to understand the prevalence of multimorbidity across WHO geographic regions among adults.

**Methods:** We performed a systematic review and meta-analysis of surveys designed to estimate the prevalence of multimorbidity among adults in community settings. We searched PubMed, ScienceDirect, and Google Scholar databases for studies published between January 1, 2000, and December 31, 2021. The random-effects model estimated the pooled proportion of multimorbidity in adults. Heterogeneity was quantified using I2 statistics. We performed subgroup analyses and sensitivity analyses based on continents, age, gender, multimorbidity definition, study periods and sample size. The study protocol was registered with PROSPERO (CRD42020150945).

**Results:** We analyzed data from 126 peer-reviewed studies that included nearly 15.4 million people (32.1% were male) with a weighted mean age of 56.94 years (standard deviation of 10.84 years) from 54 countries around the world. The overall global prevalence of multimorbidity was 37.2% (95% CI= 34.9-39.4%). South America (45.7%, 95% CI= 39.0-52.5) had the highest prevalence of multimorbidity, followed by North America (43.1%, 95% CI= 32.3-53.8%), Europe (39.2%, 95% CI=33.2-45.2%), and Asia (35%, 95% CI=31.4-38.5%). The subgroup study highlights that multimorbidity is more prevalent in females (39.4%, 95% CI=36.4-42.4%) than males (32.8%, 95% CI= 30.0-35.6%). More than half of the adult population worldwide above 60 years of age had multimorbid conditions (51.0%, 95% CI=44.1- 58.0%). Multimorbidity has become increasingly prevalent in the last two decades, while the prevalence appears to have stayed stable in the recent decade among adults globally.

**Conclusion:** The multimorbidity patterns by geographic regions, time, age, and gender suggest noticeable demographic and regional differences in the burden of multimorbidity. According to insights about prevalence among adults, priority is required for effective and integrative interventions for older adults from South America, Europe, and North America. A high prevalence of multimorbidity among adults from South America suggests immediate interventions are needed to reduce the burden of morbidity. Furthermore, the high prevalence trend in the last two decades indicates that the global burden of multimorbidity continues at the same pace.

## CORRELATION BETWEEN FIRST TRIMESTER SERUM URIC ACID AND SUBSEQUENT DEVELOPMENT OF GESTATIONAL DIABETES MELLITUS

**Figure:**
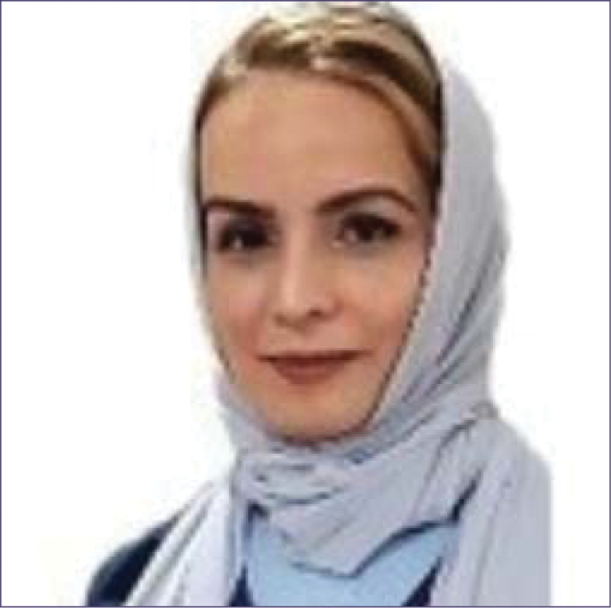


Dr. Maha Majeed

*Medical Director, Mediclinic Jowhara Hospital, Al Ain*

Dr. Doaa Taha

Dr. Sahar Mohammed Dr.Ghada Ahmed Abdel Halim

*Gestational diabetes mellitus (GDM) is the most prevalent complication encountered in pregnancy, affecting the health and well-being of millions of pregnant women all across the globe.*

**Objective:** The study’s focus was to examine the relationship between uric acid levels in the first trimester and the development of gestational diabetes mellitus subsequently.

**Methods:** The Mediclinic Jowhara Hospital in Al Ain, Abu Dhabi engaged in this prospective study. Over a period of one year from March 2022. This study comprised One hundred twenty-two prenatal women who visited the outpatient antenatal and emergency departments while in the first trimester (less than 14 weeks gestational age). Women who were fewer than 14 weeks pregnant had their serum uric acid measured, and between 24 and 28 weeks of pregnancy, they underwent an oral glucose tolerance test (OGTT) with 75 gms of glucose to be evaluated for gestational diabetes mellitus (GDM).

**Results:** In our study 86% women had normal serum uric acid value and 14% had abnormal uric acid value.

18 among the 107 women found with developed gestational diabetic mellitus, 15 had high uric acid levels and 3 had normal uric acid levels. This demonstrates that greater serum uric acid concentrations were statistically related with a higher incidence of GDM development

**Conclusions:** Though the findings of this study indicate that estimating the serum uric acid level in the first trimester may be a signal for GDM in pregnant women.

## THE ASSOCIATION BETWEEN SLEEPING BEHAVIOR, OBESITY, PSYCHOLOGICAL DEPRESSION, AND EATING HABITS AMONG ADOLESCENTS IN THE EMIRATE OF ABU DHABI – UNITED ARAB EMIRATES

**Figure:**
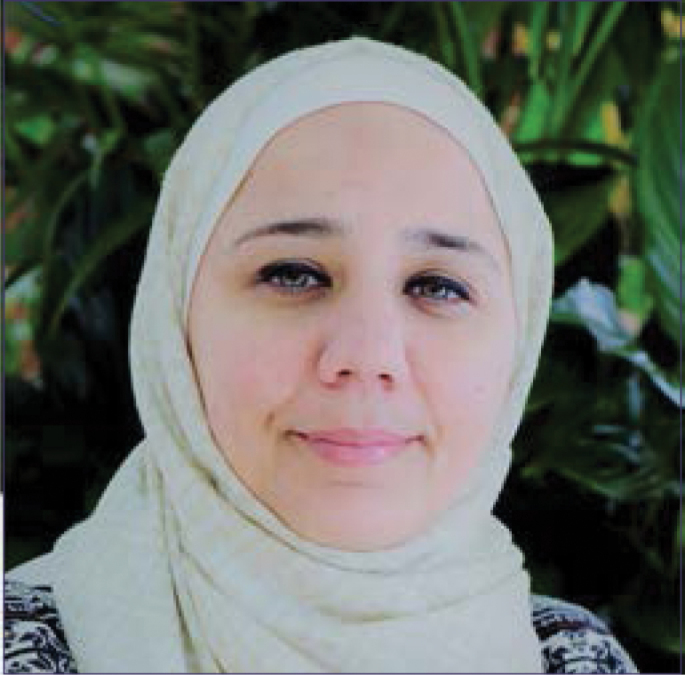


Dr. Rania Al Dweik

*Department of Public Health, Faculty of Health Science, Abu Dhabi University, Abu Dhabi*

Dr. Yousef Sheble

*School of Dentistry, Queens’ Belfast University, Belfast, UK: Hiba Ramadan Faculty of Health Science, Abu Dhabi University, Abu Dhabi:*

Haneen Issa

*Faculty of Health Science, Abu Dhabi University, Abu Dhabi*

Abdullah Sheble

*School of Medicine, Faculty of Medicine and Surgery, University of Lancashire, Preston*

**Objective:** The study aimed to investigate the association between sleeping behavior (specifically sleep duration), body mass index (BMI), eating habits, and psychological mood depression among adolescents in the Emirate of Abu Dhabi- UAE.

**Methods and Materials**:

A subsample of three hundred and eighty-five participants (209 females and 186 males) from middle and high schools (aged 12 – 18 years) in the emirate of Abu Dhabi completed the surveys in the presence of their parents and two research assistants. Measures of daytime sleepiness and other sleep parameters (sleep duration on weekdays and weekends), eating habits, and mood depression questionnaire was reported.

**Results:** Differences in BMI between males and females were statistically significant (26.02 ± 4.5 vs. 24.4 ± 4.3; p < 0.01). There was a negative linear association (p< 0.01) between the students’ BMI and the weekday/ weekend sleep duration. The average weekday and weekend sleep duration ranged from 5.7 hours (weekdays) to 9.3 hours (weekends). The study showed that an increase in BMI was correlated to mood depression (p<0.05).

**Conclusion:** The study showed a clear association between short sleep duration and obesity among adolescents in the UAE. This relationship between sleep duration and obesity is less studied and less understandable. Future research about exploring how sleeping behaviors can affect obesity during adolescence can support understanding this association and create an effective intervention.

## MINITHORACOTOMY VERSUS STERNOTOMY IN MITRAL VALVE SURGERY. META-ANALYSIS FROM RECENT MATCHED AND RANDOMIZED STUDIES

**Figure:**
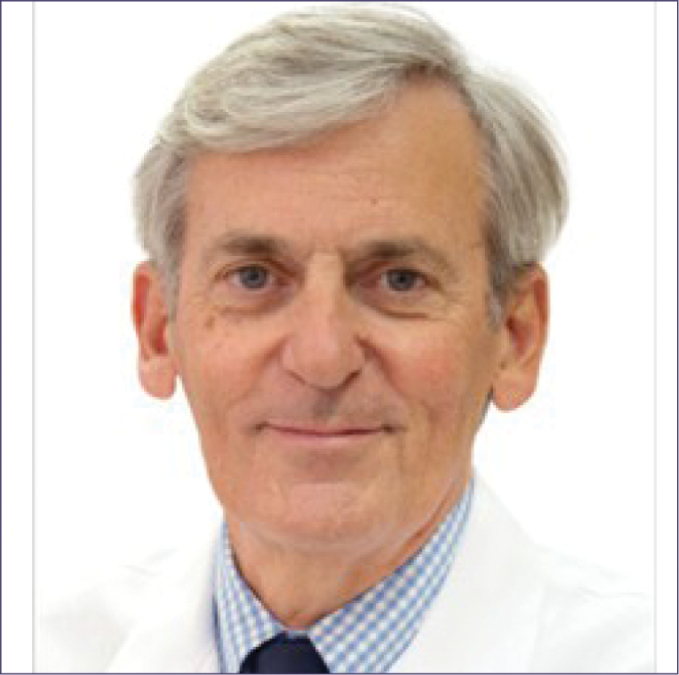


Dr. Olivier Jegaden

*Consultant Cardiologist, Mediclinic Parkview Hospital, Dubai*

Alhaitham Mahdi

*Department of cardiac surgery, Mediclinic Middle East, MBRU, AbuDhabi, UAE.*

**Objective:** Background. There is still ongoing debate about the benefits of mini-thoracotomy (MTH) approach in mitral valve surgery in comparison with complete sternotomy (STER). This study aims to update the current evidence.

**Methods:** The MEDLINE and EMBASE databases were searched through June 2022. Two randomized studies and 15 propensity score matched studies published from 2011 to 2021 were included with a total of 11561 patients operated on from 2005 (MTH: 5749, STER: 5812). Data regarding early mortality, stroke, reoperation for bleeding, new renal failure, new onset of atrial fibrillation, need of blood transfusion, prolonged ventilation, wound infection, time-related outcomes (cross clamp time, cardiopulmonary bypass time, ventilation time, length of intensive care unit stay, length of hospital stay), midterm mortality and reoperation, and costs were extracted and submitted to a meta-analysis using weighted random effects modeling.

**Results:** The incidence of early mortality, stroke, reoperation for bleeding and prolonged ventilation were similar, all in the absence of heterogeneity. However, the sub-group analysis of early mortality showed a significant OR in favor of MTH when robotic enhancement was used. New renal failure (OR: 1.62, 95% CI, 1-2.63, p=0.05), new onset of atrial fibrillation (OR: 1.28, 95% CI, 1.10-1.48, p=0.001) and the need of blood transfusion (OR: 1.77, 95% CI, 1.39-2.21, p=0.001) were significantly lower in MTH group. Regarding time-related outcomes, there was evidence for important heterogeneity of treatment effect among the studies. Operative times were longer in MTH: differences in means were 20.7 min for cross clamp time (95% CI, 14.9-26.4, p=0.001), 36.8 min for CPB time (95% CI, 29.8-43.9, p=0.001) and 37.7 min for total operative time (95% CI, 19.6-55.8, p<0.001). There was no significant difference in ventilation duration; however, the differences in means showed significantly shorter ICU stay and hospital stay after MTH compared to STER: -0.6 days (95% CI, -1.1/-0.21, p=0.001) and -1.8 days (95% CI, -2.86/-1.20, p=0.001) respectively, leading to a significant lower hospital cost after MTH compared to STER with difference in means -4528 US$ (95% CI, -8725/-326, p=0.03).

The mid-term mortality was significantly higher after STER compared to MTH: OR= 1.79, (95%CI) 1.34-2.39, p=0.001; the rate of mid-term reoperation was reported similar in MTH and STER: OR=0.84, (95%CI) 0.59-1.42, p=0.52.

**Conclusions:** The present meta-analysis confirms that the MTH approach for mitral valve disease remains associated with prolonged operative times, but it is beneficial in terms of reduced postoperative complications (renal failure, atrial fibrillation, blood transfusion, wound infection), of reduced length of stay in ICU and in hospitalization, with finally a reduction in global cost. MTH approach appears associated with a significant reduction of postoperative mortality that must be confirmed by large randomized study.

## THE ASSOCIATION BETWEEN SLEEPING BEHAVIOR, OBESITY, PSYCHOLOGICAL DEPRESSION, AND EATING HABITS AMONG ADOLESCENTS IN THE EMIRATE OF ABU DHABI – UNITED ARAB EMIRATES

**Figure:**
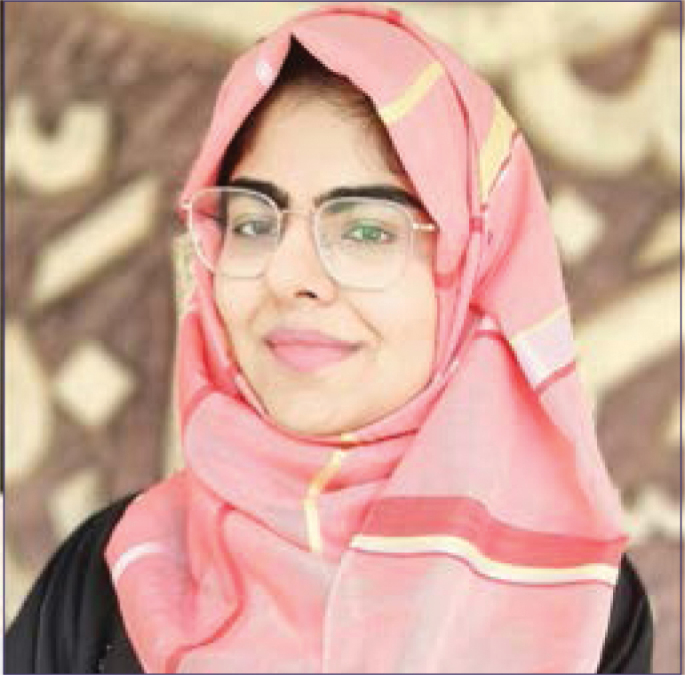


Shiza Saleem

*Research Assistant, Khalifa University, Abu Dhabi*

**Background:** The use of beta-blockers is the standard pharmacological approach used in heart failure patients. It shows promising results by reducing hospitalization rates and improving survival especially in reduced ejection fraction (HFrEF). However, the effect of the beta-blockers on heart failure with preserved ejection fraction (HFpEF) is less clear with few studies investigating this. Decreased heart rate variability (HRV) is associated with cardiac pathology including heart failure and can be used to assess cardiac health

**Objectives:** To investigate the effect of beta-blockers on patients with HFpEF for time periods of increased risk of cardiac events.

**Methods:** ECGs of seventy-three patients with HFpEF > 55% were recruited. Fifty-six patients were on only beta-blocker medication and 17 patients on no medication. HRV features were extracted from the ECG for 6 – 10 am and 6 – 10 pm hours for two and four-hour windows. Statistical analysis was performed to investigate the difference between the two groups.

**Results:** The result shows that RMSSD (p=0.011, 40%), HF power (p=0.012, 134%), and VLF power (p=0.047, 63%) were significantly higher during the 6 – 10 am interval for the beta-blocker group. Sample entropy (p=0.016, 32%), and the novel fragmentation measures PIP (p=0.015, 25%), IALS (p=0.015, 22%) and PSS (p=0.008, 22%) were significantly higher between 6 – 10 pm.

**Conclusion:** Beta-blocker therapy increases HRV measures in the HFpEF group depending on the feature investigated indicating an overall decreased risk of a cardiac event and a possibly beneficial effect of beta-blockers, especially during the morning hours that is characterized by a sympathetic surge.

## MRI-GUIDED VACUUM-ASSISTED BREAST BIOPSIES: OUR EXPERIENCE AS THE FIRST PRIVATE HEALTHCARE FACILITY SETTING UP THE SERVICE IN UAE

**Figure:**
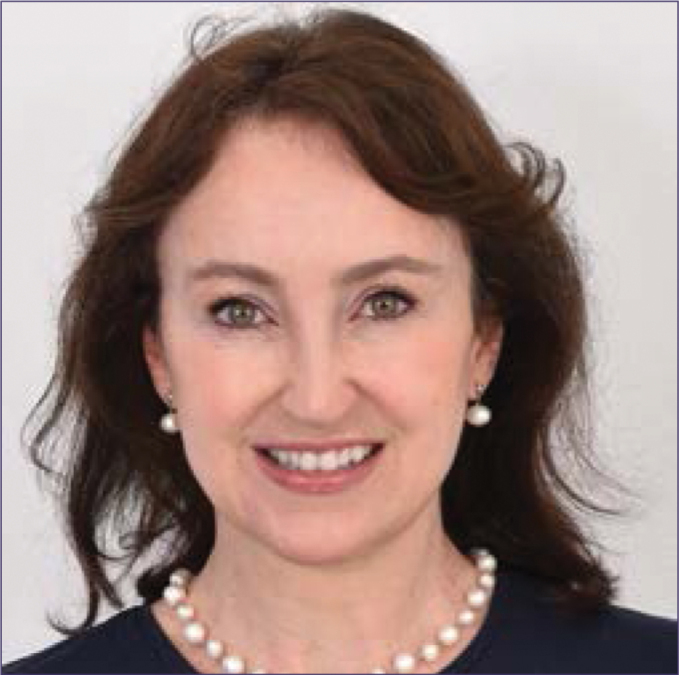


Dr. Alexandra Economacos

*Consultant Breast Radiologist, Mediclinic City Hospital, Dubai, UAE.*

Dr Leila Ismail

Dr Konstantia Diana Stavrou Dr. Yee Ting Sim

*Consultant Breast Radiologist, Mediclinic City Hospital, Dubai, UAE*

**Objective:** MRI guided vacuum biopsy-VAB-is used to diagnose the causes of indeterminate enhancing lesions identified on contrast breast MRI that cannot be further assessed under mammography or ultrasound. Our department is the first private facility to offer this option in the UAE and we wished to determine the safety and efficacy of this service for patient disease management)

**Methods:** Retrospective audit of all MRI-guided breast VAB’s in our hospital from February 2020 to September 2022 was conducted. Data collated from Radiology Information System, biopsy and surgical pathology reports from Electronic Medical Records. Excluded from the audit are patients in whom the planning MRI sequences conducted on the day of VAB no longer show abnormal enhancement.

**Results:** Total of 50 MRI-guided VAB’s were performed since inception of the service. Technical success rate is 100%. The histopathology outcomes are: benign, B2 (n = 35, 70%), lesions of uncertain malignant potential, B3 (n=9, 18%), malignant, B5 (n=5, 10%). To date, there are no major peri- or post-procedural complications requiring surgical intervention. Minor complications include haematoma (although the haematoma rate is not higher than VAB’s performed under stereotactic or ultrasound guidance), minor skin tear requiring suture.

**Conclusions:** MRI VAB is a safe and accurate method to obtain histological diagnosis of indeterminate enhancing breast lesions at our facility with limited complications. With this additional service, unnecessary extensive breast surgery has been avoided, with good patient outcomes.

**POSTER PRESENTATION ABSTRACTS**

## VARIATIONS IN IRON DEPOSITION AND IRON–REGULATING PROTEINS IN MICE WITH EXPERIMENTAL AUTOIMMUNE ENCEPHALITIS

Ahmed M. Al-Rawi

*Department of Medical Laboratory Sciences, Collage of Health Sciences, University of Sharjah, Sharjah Institute of Medical Research, Sharjah, United Arab Emirates, sahoki99@hotmail.com*

Mohammad G Mohammad*

Department of Medical Laboratory Sciences, Collage of Health Sciences, University of Sharjah, Sharjah Institute of Medical Research, Sharjah, United Arab Emirates, mmohd@sharjah.ac.ae

**Background:** Multiple sclerosis (MS) is a neurodegenerative disease characterized by chronic inflammation, associated with demyelination and axonal degeneration, as well as damage to myelin-producing oligodendrocytes. MS is increasingly linked to dysregulation of iron metabolism. Despite being a vital element in many metabolic and enzymatic activity, iron overload lead to toxic accumulation and consequential damage to neuronal cells. Pathological evidence suggests that iron regulatory proteins are altered in different tissues during the course of MS, as well as in specific anatomical areas of the brain. To further delineate the changes in iron regulation, we used mice with Experimental Autoimmune Encephalitis (EAE), a well-characterized and used model of autoimmune pathology of the CNS that resembles MS.

1. Objective: This study aimed at investigating the changes in the distribution of iron deposits in the central nervous system (CNS) and systemic compartment in EAE mice and to characterize the expression and distribution of key proteins controlling iron metabolism in mice with EAE.

**Methods:** 15 wild-type female C57BL/6 mice (~12 weeks old) were used to induce EAE. Mice were subcutaneously injected with myelin oligodendrocyte glycoprotein (MOG). Following MOG injection, mice received an intraperitoneal injection of pertussis toxin (PTX) (100 ng/120 μl) on day 0 after 1h of the MOG immunization, and on day 2. Clinical scoring of EAE mice was recorded daily by observing their motor functions. Consequentially, the mice were euthanized once they had reached the peak of the disease, and different organs were collected and stored at -80 °C, to determine iron

metabolism-regulating proteins expression. Tissue samples needed for Prussian blue staining were kept in a 10% natural formalin buffer for assessing iron deposits.

**Results:** Results of the Prussian blue staining indicated increased iron deposits in EAE mice in brain (olfactory bulb, meninges, and cerebrum), spinal cord, spleen, and liver. Iron-related proteins were altered in EAE mice in different brain areas. Iron regulatory protein (Hepcidin) levels in the olfactory bulb, spinal cord, liver, and spleen were increased in association with reduced expression of ferroprotein-iron efflux transporter. As a result, the protein responsible for iron storage (Ferritin) increased significantly in these anatomical areas, which confirms the Prussian blue staining findings. Moreover, hypoxia- inducible factor-1 (HIF-1α) an iron regulatory protein was increased in all of the different anatomical areas of EAE mice that were assessed. As a result of neuroinflammation, significant changes in iron regulatory proteins were observed in the cervical and inguinal lymph nodes in EAE mice.

**Conclusions:** Although further studies are needed to ascertain the exact phenotype and location of the iron internalizing cells within the brain and spinal cord, our results suggest that EAE induces a CNS and systemic alteration of iron metabolism which may play a role in the pathogenesis and aggressiveness of the disease. Neuroinflammation mediated by EAE resulted in variations in iron deposition and proteins controlling iron metabolism in various anatomical areas in the brain. Further investigation of the pathways mediating iron regulating proteins changes would help further understanding the neurotoxicity caused by iron depositions during EAE.

## A LEAN SIX SIGMA METHODOLOGICAL APPROACH TO REDUCE THE WAITING TIME & IMPROVE THE PATIENT FLOW WITH OPTIMIZED PATIENT EXPERIENCE IN OUT PATIENT PHYSICAL THERAPY DEPARTMENT

Albert Anand, MPT,MBA, LSSBB

*Team Lead – Physiotherapy, Mediclinic Al Noor Hospital Co Authors - (Physio Team & PA’s)*

**Background:** Physical therapy plays a vital role in the health care setting. It has becomes one of the primary areas in the health care management. Lean Six Sigma (LSS) is the latest generation of improvement approaches. The amount of waiting time directly impact in patient experiences in a physical therapy outpatient care. Patient waiting time data

supports, Patients referred to physical therapy Department for appointment waited a minimum of three weeks. The physical therapy staff found this frustrating as patient’s conditions often became more chronic during the wait and they often needed to take prolonged periods of sick leave. Physical therapists felt that in order to provide a better quality service they should reduce the waiting time of the patients.

**Objectives:** To reduce the patient waiting time & improve the patient flow with optimized patient experience to using LSS methodology in an Outpatient Physical therapy department (OPD).

**Method:** Methodology: Lean Six Sigma DMAIC (Define-Measure-Analyze-Improve-Control) methodology & using different quality tools.

Design: Descriptive Cross sectional Study. Sampling: Simple random Sampling. Duration: 6 weeks

Study Setting: MNOO- Physiotherapy

Outcome measure: Waiting time/days & VOC Survey

Outcome Tools: Patient Tracker & patient satisfaction survey questionnaire’s’ (NHS) Quality Tools: Flow Chart, VOC Analysis, SIPOC, SWOT, GEMBA & Run Chart

**Results:** A number of non-value added activities were identified within the process and actions were initiated to systematically eliminate different forms of waste using the principles of Lean thinking. The Non value added activities of Rework, Rejections, delayed approval Process leads to revenue loss were identified and eliminated from the process. As a result of this QI project, the average waiting time reduced from 13.5 days (2 weeks) to 4 days to execute the first physical therapy session from the insurance approval.

**Conclusion:** There was a significant reduction in waiting time & improving the patient flow was achieved in the outpatient services of the physical therapy department by using the lean six sigma approach.

## EFFECT OF PASSIVE STRETCHING OF RESPIRATORY MUSCLES ON CHEST EXPANSION AND SIX-MINUTEWALK DISTANCE IN COPD PATIENTS

Asma Rehman 1, Jyoti Ganai 2, Rajeev Aggarwal 3, Ahmad H. Alghadir 4 and Zaheen A. Iqbal 4,*

*1 Al Hosn One Day Surgery Center LLC, Al Sahel Tower Building, Post Box 37384, Abu Dhabi, UAE*

*2 Department of Rehabilitation Sciences, Jamia Hamdard, New Delhi 110062, India*

*3 Neuro-Physiotherapy Unit, NSC, All India Institute of Medical Sciences, New Delhi 110029, India*

*4 Rehabilitation Research Chair, College of Applied Medical Sciences, King Saud University, Riyadh 11433, Saudi Arabia*

* Correspondence: z_iqbal001@yahoo.com or zaiqbal@ksu.edu.sa

**Background:** Chronic obstructive pulmonary disease (COPD) is a major cause of morbidity and mortality worldwide. Hyperinflation of the lungs leads to a remodeling of the inspiratory muscles that causes postural deformities and more labored breathing.

Postural changes include elevated, protracted, or abducted scapulae with medially rotated humerus, and kyphosis that leads to further tightening of respiratory muscles. As the severity of the disease progresses, use of the upper limbs for functional tasks becomes difficult due to muscle stiffness. There are various studies that suggest diffierent rehabilitation programs for COPD patients; however, to the best of our knowledge none recommends passive stretching techniques.

**Aim:** Aassess the effect ofrespiratory muscle passive stretching on chest expansion and 6-min walk distance (6MWD) in patients with moderate to severe COPD. Methods: Thirty patients were divided into two groups, experimental (n = 15) and control (n = 15). The experimental group received a hot pack followed by stretching of the respiratory muscles and relaxed passive movements of the shoulder joints. The control group received a hot pack followed by relaxed passive movements of the shoulder joints. Results: In the control group, there was no difference in chest expansion at the levels of both the axilla and the xiphisternum or in 6MWD between baseline and post treatment (p > 0.05). In the experimental group, chest expansion at the level of the axilla (p < 0.05) and 6MWD (p < 0.001) were significantly higher post treatment, while there was no difference in chest expansion at the level of the xiphisternum (p > 0.05). A comparison between control and experimental groups showed that chest expansion at the level of the axilla (p < 0.05) and 6MWD (p < 0.01) were significantly higher in the experimental group, while there was no difference in chest expansion at the level of the xiphisternum (p > 0.05).

**Conclusions:** Although COPD is an irreversible disease, results of this study indicate that passive stretching of respiratory muscles can clinically improve the condition of such patients, especially in terms of chest expansion and 6MWD. Given the good effects of muscle stretching and the fact that such an exercise is harmless, clinicians and physiotherapists should consider including passive stretching of respiratory muscles in the rehabilitation plan of COPD patients.

## FETAL RIBS; SPECTRUM OF ABNORMALITIES

Bernard Nasr MD.

*Mediclinic Airport Road Abu Dhabi. Fetal Medicine unit, OBGYN department. Bernard.Nasr@mediclinic.ae*

**Muzibunissa Began MD.**

*Mediclinic Airport Road Abu Dhabi. Fetal Medicine unit, OBGYN department. Muzibunissa.Begam@mediclinic.ae*

**Feroza Dawood MD.**

*Mediclinic Airport Road Abu Dhabi., OBGYN department. Feroza.Dawood@mediclinic.ae*

**Objective:** The purpose of this study is to describe the clinical importance of an abnormal number and shape of fetal ribs.

**Methods:** A retrospective study of fetuses that were found to have an abnormal number and/or aspect of their ribs during routine ultrasonographic examinations, using static 3- or 4-Dimensional volume between 2018-2021. In all cases, a meticulous survey of the fetal anatomy was performed, and prenatal and postnatal records were reviewed.

**Results:** 24 fetuses were found retrospectively to have an abnormal ribs count or shape. Ultrasonographic examinations were done between 16 and 31 weeks’ gestation (median, 22.1weeks). More than 24 ribs were found in 11 fetuses, and fewer than 24 ribs were found in 4 without associated chromosomal abnormality. 5 fetuses had signs of achondrodysplasia, 2 with features of osteogenesis imperfecta and 2 with Short Rib Polydactylia Syndrome. Additional anomalies were found in 4 fetuses (Small VSD, thick nuchal fold, pyelectasis, short femur).

**Conclusion:** The analysis and numeration of fetal ribs should be done routinely. Ribs abnormalities may be the first manifestation of many congenital conditions.

## ASSESSMENT OF PATIENT SATISFACTION WITH PERIOPERATIVE ANESTHESIA CARE AT MEDICLINIC PARKVIEW HOSPITAL: A CROSS SECTIONAL STUDY IN DUBAI, UNITED ARAB EMIRATES.

Contact author: Dr Indira Kannan

*Consultant Anaesthetist -Mediclinic Parkview Hospital, Dubai; Adjunct Clinical Assistant Professor- MBRU, Dubai; Indira. Kannan@mediclinic.ae.*

**Second author: Saher Bhati**

*College of Medicine, Mohammed Bin Rashid University of Medicine and Health Sciences, Dubai, U.A.E Saher.bhati@students.mbru.ac.ae*

**Second author: Fatma Abdulle**

*College of Medicine, Mohammed Bin Rashid University of Medicine and Health Sciences, Dubai, U.A.E Fatma.abdulle@students.mbru.ac.ae*

**Objective:** This study aimed to assess the overall patient satisfaction with perioperative anaesthesia care and determine the factors that influence it at Mediclinic Parkview Hospital, Dubai.

**Methods:** A prospective cross-sectional study will be conducted between October 2022 and May 2023. All patients undergoing elective surgeries will receive an electronic questionnaire postoperatively. Patient satisfaction will be determined by a five-point Likert scale. Patient demographics and data regarding different dimensions of the perioperative experience (information received from the anaesthesia team, patients’ impression of the anaesthesia team, patients’ discomforts, and patients’ feelings) was recorded. Descriptive and inferential analysis was conducted on the data using Statistical Package for Social Sciences Windows version 26. Statistical significance was set at P < 0.05.

**Results**

The following describes the study’s preliminary results from the first two weeks of October 2022. Thirteen of the twenty- six patients who received the questionnaire responded. The overall patient satisfaction was 96%. Postoperative pain (38.5%) was the leading cause of patient discomfort, followed by hunger (30.8%). Patients with a better impression of their anaesthesia team and satisfactory control of postoperative symptoms had a significantly higher overall satisfaction score (P < 0.05).

**Conclusion:** Patients’ impression of their anaesthetic team and the control of postoperative symptoms were major determinants of their overall satisfaction. As the first study of its kind in the U.A.E, it can help enhance the quality of anaesthetic care in the country by focusing on these factors to yield a more patient-centric perioperative experience.

## IMPACT OF ANTIBIOTIC STEWARDSHIP PROGRAM (ASP) ON SURGICAL ANTIBIOTIC PROPHYLAXIS COMPLIANCE AND RESTRICTED LIST OF BROAD SPECTRUM ANTIBIOTIC USAGE

Dr Gihan Kamal Madbouly,

*Clinical Pharmacist, Pharmacy Department, Mediclinic AL Noor Hospital, Abu Dhabi, UAE*

**Background:** The overuse and misuse of antibiotics has resulted in the growth of super-bugs that are increasingly resistant to available antibiotics. According to the US Centers for Disease Control and Prevention (CDC), drug-resistant bacteria cause 23,000 deaths and 2 million illnesses each year. The Institute for Healthcare Improvement reported that 25,000 people die each year in Europe from antimicrobial resistance, and microbial resistance is growing in the Middle East, Africa, and Asia. In order to reduce the development and spread of resistant bacteria and deliver better patient outcomes, healthcare facilities must implement measures to ensure optimal use of antibiotics.

Implementation of an antibiotic stewardship program (ASP) will help healthcare facilities reach the goal of providing patients requiring antibiotic treatment with the right antibiotics, at the right time, at the right dose, and for the right duration. This can be through the selection of appropriate prophylactic antibiotic, timing of preoperative antibiotic administration and duration of postoperative prophylactic antibiotic. Moreover, restricting the use of broad spectrum antimicrobials by ensuring their use when necessary indicated shall help in decreasing the resistance pattern of these antibiotics.

**Objectives:** ASP goals shall monitor the compliance for the below elements:

• Selection of prophylactic antibiotic as per Mediclinic Middle East (MCME) guidelines

• Discontinuation of postoperative prophylactic Antibiotic within 24 hours after surgery Preoperative Antibiotic Administration timing within 60 minutes

• Number of Defined Daily Dose (DDD) per 100 patient admissions and Antibiotic consumption use/cost are calculated for interpretation semiannually.

**Method:** ASP Committee members (Internal medicine specialist, Clinical Microbiologist, Clinical Pharmacist, Infection Control officer, General Surgeon and Quality/patient Safety Officer) conducted monthly monitoring in 2021 and 2022 through a retrospective audit of all the patients who had surgeries within the hospital. Monitoring of Restricted list usage of broad spectrum antibiotics were conducted through validating the preauthorization approval form of the list, appropriate indication and duration.

Continuous awareness and education was continuously emphasized by the team to all healthcare professionals by sharing the main focus elements of ASP including Surgical Antibiotic Prophylaxis guidelines and the restricted list of broad spectrum antibiotic appropriate usage that requires preauthorization approval.

A defined compliance target was agreed for three KPIs (98%) which are the selection of preoperative prophylactic antibiotic according to MCME guidelines, discontinuation of postoperative prophylactic antibiotic within 24 hours and timing of preoperative prophylactic antibiotic administration within 60 minutes. Number of DDD per 100 patient admissions and Antibiotic consumption use/cost were calculated to assess improvement and performance semiannually.

**Results:** • Average compliance of Prep-op antibiotic surgical prophylaxis in 2022 (Q1&Q2) is 95% compared to 94% in 2021. Average compliance of Post-op antibiotic surgical prophylaxis in 2022 (Q1&Q2) is 98% compared to 97% in 2021. Average compliance for pre-op antibiotic administration timing within 60 minutes was 96.2% in 2021 & 2022.

• Restricted list of antibiotics/antifungals showed an improvement (Q1/Q2 2022) in all antibiotic/antifungal compared with (Q1/Q2 2021) by achieveing a decrease in the number of DDD per 100 patient admissions compared with 2021 except for Meropenem.

• Antibiotic Consumption use and Cost for restricted list of broad spectrum antibiotics showed a decrease in consumption and cost reduction for all antibiotics in 2022 except Meropenem compared with 2021.

• Rational is attributed to lack of awareness and education about ASP program especially for new staff and increase in 2022 admissions of Post COVID-19 patients which indicated the use of broad spectrum antibiotic like Meropenem.

**Conclusion:** Implementation of ASP has a significant positive impact on compliance with Surgical Antibiotic Prophylaxis Guidelines and decrease in number of DDD/Consumption and Cost of Broad Spectrum Antibiotics. Continuous monitoring and raising healthcare professional awareness is required for further improvement.

## ANTI-CANCER POTENTIAL OF FRONDANOLTM: A NUTRACEUTICAL EXTRACT FROM CUCUMARIA FRONDOSA

Hardik Ghelani

*College of medicine, Mohammed Bin Rashid University Of Medicine and Health Sciences, Dubai, United Arab Emirates. (Hardik.ghelani@mbru.ac.ae)*

**Md Khursheed1, Peter Collin2, Thomas Edward Adrian1, Reem Kais Jan1**

*1College of Medicine, Mohammed Bin Rashid University of Medicine and Health Sciences, Dubai, United Arab Emirates.*

*2Coastside Bio Resources, Stonington, ME, USA*

*md.khursheed@mbru.ac.ae (M.K.); thomas.adrian@mbru.ac.ae (T.E.A.); peter.d.collin48@gmail.com (P.C.); reem.jan@ mbru.ac.ae (R.K.J.)*

**Objective:** Frondanol is a nutraceutical lipid extract of the edible Atlantic sea cucumber, Cucumaria frondosa. It has been shown to have significant anti-inflammatory activity in the adjuvant arthritis rat model and ear edema mice model. However, to date, the anticancer activity of Frondanol has not been explored. Although the mechanism of action of Frondanol is not known yet, it is thought to inhibit both 5-lipoxygenase (LOX) and 12-LOX pathways, suppressing the production of 12-hydroxyeicosatetraenoic acid, 5-hydroxyeicosatetraenoic acid, and leukotriene B4 in human polymorphonuclear cells. Interestingly, previous studies have shown that 5-LOX plays a critical role in arachidonic acid-stimulated cancer cell growth. Further exploration of 5-LOX enzyme activity inhibition by Frondanol may have valuable therapeutic applications in the treatment of human cancer.

**Methods:** In this study, we investigated the effects of Frondanol on cell growth and apoptosis in HT-29 and Caco-2 colorectal cancer cells. Cell growth and apoptosis were evaluated by cell cycle analysis (late apoptotic event) and by annexin assay (early apoptotic event), respectively.

**Results:** Frondanol inhibited cell growth of HT-29 and Caco-2 cells within IC50 values of 0.09% and 0.07 % (v/v) at 24 h, respectively, and 0.05% v/v and 0.04% (v/v) at 48 h, respectively. The annexin assay results showed early induction of apoptosis in HT-29 cells by Frondanol.

**Conclusion:** Frondanol inhibited cell growth and induced early apoptosis in colorectal cancer cells, possibly through the inhibition of 5-LOX enzyme activity. Further experiments including cell cycle studies and 5-LOX enzyme inhibition activity are currently underway.

## NURSE REPORTED PERCEPTIONS OF MEDICATION SAFETY IN PRIVATE HOSPITALS IN GAUTENG PROVINCE

Madré Paarlberg

*North West University, South Africa drakienel@gmail.com*

Dr Alwiena J Blignaut

*North West University, South Africa alwiena.blignaut@nwu.ac.za*

**ABSTRACT**

Medication administration errors remains a global patient safety problem targeted by the WHO, yet research on this matter is sparce within the South African context.

**Objective:** To explore and describe private sector nurses’ perceptions on medication administration safety-related culture, incidence, causes, and reporting in the Gauteng Province.

**Methods:** Online surveys were utilized (N=768, n=217) in a quantitative research design. SPSS was used for descriptive and inferential data analysis.

**Results:** Although teamwork within units (M=3.58; SD=0.89) were deemed satisfactory, a punitive response to errors (M=3.14; SD=0.96) was highlighted. 70.7% (n=152) of respondents reported working in “crisis mode” whereas 61.6% (n=133) worried that their mistakes were recorded. More than half of the respondents (57.7%, n=124) reported long working hours as impacting patient safety. Most respondents graded overall medication safety positively (n=209, 96.4%) with the exception perceiving medication administration errors a daily problem (3.6%, n=8). Work overload (M=3.38; SD=0.80), high patient-nurse ratios (M=3.28; SD=0.90), and inadequate staffing (M=3.25; SD=0.84) were implicated most as error- inducing. Medication administration errors were reported most of the time (M=3.76; SD=1.06), irrespective of harm being caused or not. Fear (M=3.80; SD=1.62) and administrative response to errors (M=3.70; SD=1.40) were the major reasons of non-report. Correlations suggested that reasons for non-report of errors were affected by non-punitive safety culture (r=0.422-0.466, p<0.001).

**Conclusions:** Medication administration safety improvement is contingent on fostering a non-punitive safety culture within units. Anonymous medication error reporting systems and auditing nurses’ workload are recommended in the quest of improved medication safety within Gauteng Province private hospitals.

## A SINGLE INSTITUTION EXPERIENCE OF ROBOTIC TAPP: FIRST 136 CASES

Dr Maliha Jaffar, Dr Muhammad Umar Younis, Dr Faisel Ikram, Dr Hemant Vadeyar and Dr Roger Gerjy

*Department of General Surgery, Mediclinic City Hospital Dubai, UAE*

**Objective:** Inguinal hernia repair is a surgery to repair a hernia in the abdominal wall of the groin. A hernia is tissue that bulges out of a weak place in the abdominal wall. In some cases, the intestines may also bulge out in the weakened area.

A robotic-assisted inguinal hernia repair is an ideal procedure for patients who have hernia on both sides of their groin. Since one of the incisions is made through the belly button, patients who also have umbilical hernias can have a repair done at the same time.

During this procedure, the surgeon places three ports, one 8 mm at the belly button and two 8 mm ports on each side of the lower abdomen. Since the robotic instruments are flexible and wristed, dissection in the ceiling of the anterior abdominal wall where the hernias are can be done with ease. During the hernia repair, the bulging tissue is pushed back in. A special 3D Mesh is placed through the port and this helps the abdominal wall regain strength.

Robotic surgery adds high definition visualization and since the robotic arms are wristed, the Mesh is placed easily and recovery time is minimal compared to traditional open hernia repair and much less pain and earlier return to normal activity, comparable recurrence rates, cost effectiveness, and a notable decrease in wound-related problems. We describe here our early experience of adoption of DaVinci Xi Robot for Inguinal Hernia Repairs. During an operation, the surgeon sits at a console in the surgical suite and directs the robotic arms to perform the surgery. The robot seamlessly and directly translates the surgeon’s natural hand, wrist and finger movements from controls at the console to the surgical instruments inside the patient.

**Methods:** A retrospective analysis of a single institution database was carried out and all robotic inguinal hernia repairs using the Transabdominal Preperitoneal approach (TAPP) from June 2020 to June 2022 were scrutinized. The data parameters collected constituted patient demographics (age, gender), intraoperative variables (docking and console times) and postoperative results (length of stay and early complications).

**Results:** Our initial findings indicated a total of 136 patients that underwent robotic repair of inguinal hernia with 98.5% of patients being male (134 male) and only 2 patients being female. Mean age at operation was 47.96 years. Intraoperative times showed a downward as the robotic team became more proficient in the procedure. Docking times improved with a mean of 4.82 min (1-10) to 2.88 min (1-7) while the console times improved from a mean of 48.54 min (18-138) to merely 46.09 min (18-99). Mean length of stay of patients was 1.07 days (Range 1-4). No complications were encountered and early results seemed promising on routine follow up.

**Conclusion:** Our initial experience upholds the superiority of Robotic repair of inguinal hernia with excellent results. Although it remains technically challenging, requiring advanced skills, learning curve and familiarity with the approach, but better robotic-assisted visualization, dexterity, precision and control, that a surgeon can perform a wide array of procedures through small incision is the rationale behind pursuing its implementation and perfection.

## NEUROLOGICAL MANIFESTATIONS AFFLICTED BY COVID-19: A COHORT STUDY

Mallika Mehrotra

*MBRU: Mallika.Mehrotra@students.mbru.ac.ae*

Swetha Lekshmi

*MBRU: Swetha.Lekshmi@students.mbru.ac.ae*

**ABSTRACT**

SARS-CoV-2 has caused a worldwide pandemic due to its high transmission rate amongst humans and causes a threat to the global health. Numerous recent research suggest that the SARS-CoV-2 has detrimental effects on the brain processes and may even cause major neurological impairment during this continuing contagion. Some of the main neuroinvasion pathways through which SARS-CoV-2 can cause neurologic events are direct invasion, hematogenic routes, hypoxia, and cytokine storm.

**Methods:** In the current work, we collected the data of 473 COVID-19 patients from the Baynaty database and found 44 COVID-19 patients who had neurological consequences.

**Objectives:** We detailed the potential pathways for SARS-CoV-2 entry into the nervous system, the subtypes of neurological manifestations, the underlying comorbidities and key variables that induced them. The central nervous system (CNS) effects of the SARS-CoV-2 infection are evaluated with regards to their neurological manifestations.

**Results:** Through our data analysis we found that majority of the patients who were afflicted by such neurologic events had a mean age of 64 years and had associated comorbidities such as diabetes, hypertension and cardiovascular diseases. The most common neurologic subtype was ischemic stroke, and the most common comorbidity was hypertension.

**Conclusion:** This research can help healthcare workers prioritize COVID-19 patients with such comorbidities and take preventive measures to ensure the patient doesn’t develop neurological manifestations.

## THE USE OF ARTIFICIAL INTELLIGENCE IN COLONOSCOPY IMPROVES ADENOMA DETECTION RATES AND INVERSELY REDUCES THE RISK OF INTERVAL COLORECTAL CANCER; FIRST COMPARATIVE STUDY IN UAE;

Mazin Aljabiri, Usama Warshow Alexandra Deduchova, Rola Saadi, Ammar Al Hassan, Joyce Villanueva, Jam Tomagan, Archebal Alimagno, Liji George, Saranya Das, Joven Dadang, Kamille Paz, Allysa Badong, Antonette Santos.

*Mediclinic Parkview Hospital United Arab Emirates-Dubai*

**Background:** Globally, colorectal cancer (CRC) is the third most commonly diagnosed cancer in males and the second in females, with the second-highest cancer mortality rate(1). Adenomas are a major precursor lesions for CRC and as such adenoma detection rate (ADR) is an important and well recognized quality indicator of colonoscopy-based screening & surveillance worldwide. ADR is defined as the fraction of patients undergoing first-time screening colonoscopy who have one or more conventional adenomas detected and identified by pathology. The recognised minimum quality standard in a mixed-gender screening population is 25%. It is estimated that for every 1% increase in ADR, a patient’s risk of developing colon cancer over the next year decreases by 3%(2). Therefore, recent evidence suggestions have aspired to ADR threshold up to 39-50% to provide increased protection against post-colonoscopy interval cancer occurrence(3,4,5) Furthermore, raising the ADR leads also to reduction in Adenoma Miss Rate (AMR) (6), Artificial intelligence-assisted colonoscopy (AIAC) has gained attention as a tool to assist with polyp detection during colonoscopy(6). Artificial intelligence-assisted colonoscopy (AIAC) systems are intended to address the issue of missed polyps during colonoscopy. The effect of AIAC on ADR during screening and surveillance colonoscopy has not previously been studied in United Arab Emirates (UAE).

**Aim:** To assess and compare, for the first time in UAE, the use AIAC and its effect ADR in patients undergoing screening or surveillance colonoscopy.

**Method:** A single-centre study at Mediclinic Parkview Hospital, Dubai, UAE. The AIAC system module was utilized by five experienced endoscopists. Outcomes of consecutive surveillance colonoscopies performed for the period Apr-Dec 2020 without the use of AI & Following introduction of AI for the period Jan-Sept 2022.Comparative analysis was carried out between the two cohorts, in particular ADR. Our centre ADR was consistently above 20% over the last 10 years

**Results:** Males 51%, females 49%.indications for colonoscopy were PR bleeding, Change in bowel habits, weight loss and surveillance for FHx of polyps/cancer or abnormal imaging. A total of AIAC prospectively were compared with 666 from retrospective cohort with AI unaided colonoscopies for the period between Apr –Dec 2020 & 858 AIAC procedures. The overall polyp detection rate (PDR) was r between groups (392 vs 640); the chi-square statistic is 7.7532. The p-value is 0.005362. Significant at p <.05.

The adenoma detection rate was significantly higher in the AIAC group compared to the and Unassisted colonoscopy is (31.25% vs 23.1%)

**Conclusions:** Our Study demonstrates that AIAC resulted in a statistically significant increase in ADR (8.15%) with Prior to AI ADR detection was 23% and Post introduction of AI in colonoscopy increase to 31.25%, demonstrating the value of AIAC in a real-life cohort as ADR is an established performance indicator in colonoscopy and are inversely associated with the risks of interval colorectal cancer.(7). We recommend that all public and private sector hospitals consider implementing AIAC to improve quality of colonoscopy and optimise ADR.

## ASSESSMENT OF AN EFFECTIVENESS AND SAFETY OF ORAL MIDAZOLAM VERSUS MIDAZOLAM/ PROMETHAZINE IN UNCOOPERATIVE DENTAL PATIENTS.: A RANDOMIZED CLINICAL TRIAL.

Mohammad Thlijan*, Shaza kochaji**.

*Ph.D. in pediatric dentistry- Faculty of Dentistry- Damascus University.

**. Prof. Department of pedodontics, Faculty of dentistry, Damascus University.

**Objective:** The purpose of this study was to compare the effectiveness, safety and complications of oral midazolam alone versus midazolam/ promethazine in the pediatric dental patients.

**Materials and Methods:** Randomized clinical trial was designed and 50 children aged 3-6 years with ASA I status were selected.All the children definitely negative according to Frankle classification of child behavior.The children were divided randomly into two groups.Group 1: received oral midazolam 0.5 mg/kg.Group2: received a combination of 0.25 mg/kg midazolam and 1.1 mg/kg promethazine (orally).The vital signs were monitored befor, during an after the dental treatment.The level of sedation was measured according to UMSS scale and the chlid’s behavior was recorded by OSUBR scale.The success or failure of sedative regimen was evaulated accordind to overall behavior Houpt scale.The complications were recorded during and after dental treatment. Data was analyzed using ANOVA,Benferroni,Kruskal-Wallis and Mann-Whitney U tests.

**Results:** Patients who received 0.25mg/kg midazolame with 1.1mg/kg promethzine significantly behaved better during treatment than the placebo controls (Midazolame alone) (P<0.05). In comparison with the placebo group, reduced movement and crying were observed in the midazolam group (P<0.05). No adverse effects were observed and treatments were completed successfully.5 cases of vomiting were recorded in the control group(midazolam alone).

**Conclusion:** Midazolam/promethzine showed a significantly higher sedative effect than midazolam alone in this study in uncooperative dental children and this combination prevent vomiting and nausea.

## EFFECTIVENESS OF PERCUTANEOUS CORONARY INTERVENTION: WHAT DOES THE AUTONOMIC NERVOUS SYSTEMS HAVE TO DO WITH IT?

M. Andron, A. Qureshi, H.F. Jelinek,

*Contact Author: Dr Mohammed Andron, Consultant Interventional Cardiologist Mediclinic Airport Road Hospital, Abu Dhabi mohammed.andron@mediclinic.ae*

Second Author: DR Herbert Jelinek, Associate Professor

*Khalifa University, Abu Dhabi, herbert.jelinek@ku.ac.ae*

**Background:** In the United Arab Emirates (UAE), cardiovascular diseases (CVDs) are the leading cause of mortality, with the incidence of premature coronary artery diseases (CAD) being about 10-15 years earlier compared to developed nations. Outcomes of PCI have improved because of advancements in equipment and techniques. Never-the-less whether there is a reduction in fatal and nonfatal MI, in CAD presentations following PCI is currently still under discussion, which suggests that further subclusters (phenotypes) of patients exist. Heart rate variability (HRV) has been shown to be a strong indicator of CAD and risk of nonfatal or fatal myocardial infarct. The current study therefore investigates the changes in cardiac rhythm as a factor in assessment of PCI outcomes and possible factors in post-PCI recovery.

**Methods:** Suitable patients were selected at the MCME cardiology department for PCI. Pre-PCI and immediate post-PCI (up to 4 hours), 5-minute long heart rate recordings were obtained and analyzed for 5 HRV features using Kubios software. All statistical analyses were undertaken with EXCEL and significance set at p<0.05 for a one-way Student T-test.

**Results:** Fifteen patients had pre and post PCI results. The patient group showed bimodal distribution necessitating pre- processing. Generally, Poincare HRV dynamics increased significantly post-PCI with a concomitant significant decrease in other HRV (RMSSD, LF, HF, DFAα1, DFAα2) features. This suggests that recovery of cardiac function may be seen within the first 4 hours post PCI in select patients.

**Conclusion:** The results indicate the complex interaction of the modulation of the heart and interpretation of HRV. However, HRV feature range was correlated with parasympathetic function that differed between possible patient clusters. This latter finding requires further investigation with respect to ANS (Autonomic nervous system) modulation of post-PCI and identification of specific phenotypes that can lead to improved outcome.

## IMPROVING PATIENT SATISFACTION WITH INPATIENT FOODSERVICE: A QUALITY IMPROVEMENT STUDY IN A TERTIARY CARE HOSPITAL, DUBAI 2021-2023

Nagam Alshehabi1, Ameera Alghafri1, Montaser Al Smady1, Marwa Mohamed1, Aaesha Alshehhi2, Amera Verghese2, Eleonor Barrion2, Samuel Ho1,3, Ibtisam Alhaj2

*1Mohammed Bin Rashid University for Medicine and Health Sciences, 2Mediclinic Welcare Hospital and 3Mediclinic City Hospital*

Contact author’s name: NAGAM ALSHEHABI

*Mohammed Bin Rashid University for Medicine and Health Sciences; Nagam.Alshehabi@students.mbru.ac.ae*

Second author’s name: DR. IBTESAM ALHAJ

*Mediclinic Welcare Hospital; Ibtesam.Alhaj@mediclinic.ae*

**Abstract**

Patient experience is a growing focus for hospital strategies internationally under quality improvement initiatives. Hospital foodservice significantly contributes to patients’ overall satisfaction with inpatient care, clinical outcomes, and nutritional status. Nevertheless, foodservice satisfaction remains a complex construct with many dimensions influencing patient expectations and perceptions of the “meal experience”.

**Objective:** This quality improvement project aims to increase patient satisfaction with inpatient foodservice in a tertiary care hospital measured by Press Ganey patient satisfaction survey, and other locally applied surveys. It secondarily aims to increase nutrition referrals and decrease food-related complaints.

**Methods:** Catering process-mapping and root-cause analysis were conducted to highlight foodservice gaps. The study has a pre-post intervention design with baseline data collected from April-2021 till October-2022 guiding the FOCUS-PDCA cycle. Targeted interventions were implemented during November-2022. Post-implementation analysis is between November-2022 and February-2023 using descriptive statistics and univariate odds-ratios.

**Results:** Baseline data indicated that all Press Ganey foodservice domains were below the target (84%), except for “Courtesy”. The lowest scores included: “Food Quality”, “Getting Food Checked off Menu” and “Special Diet Explained”. Month- to-month Standard-Deviation in survey results was below 10%. Nutrition referrals were below target (60%). Post- interventional data analysis is from November-2022 to February-2023.

**Conclusions:** There is inadequate experience with quality improvement of hospital foodservice in the Middle East and worldwide. This study explores the multidimensions impacting patient satisfaction and provides qualitative analysis of patient foodservice-related concerns. Furthermore, it introduces sustainable interventions that are applicable in similar settings regionally and worldwide to optimize patient satisfaction and patient care.

## TOWARDS THE UPSCALING OF SCHOOL NUTRITION PROGRAMS IN DUBAI: AN EXPLORATORY STUDY

M. Andron, A. Qureshi, H.F. Jelinek,

*Contact Author: Dr Mohammed Andron, Consultant Interventional Cardiologist Mediclinic Airport Road Hospital, Abu Dhabi mohammed.andron@mediclinic.ae*

Second Author: DR Herbert Jelinek, Associate Professor

*Khalifa University, Abu Dhabi, herbert.jelinek@ku.ac.ae*

**Background:** School nutrition programs impact the intellectual, social, and emotional development of school children, as well as their future risk of developing Non-Communicable Diseases. While many stakeholders are involved in the development, implementation, and evaluation of school nutrition programs in Dubai, United Arab Emirates, little is known about the complementarity among those stakeholders, and the means to upscale school nutrition programs while ensuring effective, efficient, and equitable implementation. Accordingly, this study aims at exploring the perceptions of a diverse group of stakeholders, positioned at differing levels of the public health and education ecosystems in the United Arab Emirates, in relation to current guidelines and practices around the planning, implementation, and evaluation of school nutrition programs in Dubai, United Arab Emirates.

**Methods:** The study relied on a qualitative design, based on semi-structured key informant interviews. A total of 29 interviews were carried-out. Those interviewees included leaders and directors from different institutions, decision- and policy- makers, nutritionists, school nurses and nurse managers, and school principals and vice principals. All stakeholders were interviewed by the research team. Data was transcribed, and then thematically analyzed using the health systems’ model as an analytic framework.

**Results:** The thematic analysis of interview data identified five interrelated themes. The first theme relates to the limited coordination across regulatory local and federal entities, and the multiplicity of guidelines issued by the different stakeholders. The challenges around the human and financial resourcing of school nutrition programs constituted the second theme. The third theme was the weakly coordinated implementation efforts. The fourth theme was the need for better performance measurement, and the fifth theme flagged the need for improved inclusiveness for health needs and cultural preferences of the diverse student body in Dubai.

**Conclusion:** This study emphasizes that all the involved stakeholders need to better collaborate to upscale the school nutrition program in Dubai. This will require the formation of a unified governing body, which would identify and develop a single stream of resources, and sets in place a reliable, all encapsulating and equitable implementation plan along with an overarching monitoring and evaluation framework.

## NON-INVASIVE PRENATAL TESTING (NIPT) FOR ANEUPLOIDIES-A RELIABLE ACCURATE (CE-IVD) DIAGNOSIS USING WHOLE GENOME SEQUENCING (RAPID) METHOD

Priyanka Tripathi

*BTECH Biotechnology. Senior Technologist-Precision Medicine, Mediclinic City Hospital, Dubai, UAE.*

**Background:** Fetal chromosome abnormalities, specifically aneuploidies are a common cause of reproductive failure, congenital anomalies, developmental delay, and intellectual disabilities. This is are view of the ongoing non-invasive prenatal screening for pregnant women in our center.

**Aims:** The aim of this study is to bring awareness among pregnant women and to understand their preferences for risk information and decision-making concerning prenatal examinations with emphasis on NIPT. It will result in safer prenatal testing by reducing the number of invasive tests required.

**Method:** The 17 month records of all pregnant women of at least 10 weeks gestation tested for NIPT in Mediclinic City Hospital were retrospectively reviewed. NIPT screening includes common aneuploidies (chromosomes 21, 18, 13), all rare autosomal aneuploidies (RAAs), sex chromosome aneuploidies (SCAs), and partial deletions and duplications (CNVs). The pregnant women in this study were helpful for this evaluation. We generated 17 months NIPT data from different pregnant women’s across UAE, which was evaluated based on different factors affecting NIPT results like advance maternal age, maternal weight, gestational age and %ff(fetal fraction) etc.

**Results:** 1244 pregnant women were tested over the study period. Among total pregnant women 1.21% women were tested positive, where the majority was Down syndrome(+21).

80% of these positive women were advanced maternal age (≥35). In these high risk pregnancies the lowest %ff detected was 4%in positive women and 2% in normal pregnancies.

Among all positive cases 0.80% were Down syndrome (+21), 0.24% Edwards syndrome(+18) and 0.16% were Turner syndrome(X0). NIPT can tell you whether your baby is at risk for certain genetic conditions and decide whether you want it.

**Conclusions:** Women are considered high-risk if they are over 35, or if they have received a positive result from a different screening test. NIPT is quite accurate in that case. The results of this study will make a significant contribution to policy decisions and implementation of NIPT for aneuploidies within the UAE. It will result in safer prenatal testing for aneuploidies by reducing the number of invasive tests required.

## PERSISTENT SYMPTOMS FOLLOWING RECOVERY FROM COVID-19: A PROSPECTIVE CASE-CONTROL STUDY.

Duvuru R*, AlAwadhi A*, Halabi M*, HajiJama S, Elsheikh A, AlZaabi S, Suresh S, Akram S, Iqbal A, Safi AM, Narayanan NN, Aleabova S, Elsheikh AM, Balila M, Malik Z, Khamis AH, Ho SB, and Patkar S.

*Mediclinic City Hospital and Mohammed Bin Rashid University of Medicine and Health Sciences, Dubai, UAE.*

*Contributed equally

**Abstract**

Persisting symptoms greater than 3 months following COVID-19 infection have been widely reported. Most studies to date include follow up less than one year, no control group, and from a limited number of countries.

**Objective:** To determine the long-term symptoms following COVID vs non-COVID respiratory infections in Dubai, UAE.

**Methods:** Cross-sectional case control study of patients diagnosed with COVID and non-COVID respiratory infections at Mediclinic City Hospital from January 2020 to June 2021. Patient data and index infection severity obtained from medical records and patients completed a symptom questionnaire.

**Results:** 66 patients with COVID and 55 non-COVID patients completed the survey an average of 14.9 months following the index infection. COVID vs non-COVID were similar in age (mean 47.30 vs 47.75 years), gender (65% male vs 57% male) and mean number comorbidities (1.2 vs 1.18). Severity of initial infection in COVID vs non-COVID was mild-moderate (42.9% vs 55.3%) and severe (hospitalized) (57.1% vs 44.7%). Persisting symptoms at the time of the survey were present in 17 (25.7%) COVID and in 12 (21.8%) non-COVID patients. COVID patients reported 52 different symptoms (3.0/patient) and non-COVID patients reported 29 different symptoms (2.41/patient). These symptoms were considered severe in 29.4% COVID and 16.6% non-COVID patients. The most common symptoms were cardiorespiratory (52.9%), cognitive (35.3%), and headache (17.6%) in COVID patients and cardiorespiratory (58.3%), headache (25.0%) and fatigue (16.6%) in non- COVID patients. Patients with prior severe index infections tended to be more likely to have persisting symptoms in both groups.

**Conclusion:** Long-term (over one year) persisting symptoms are reported following both COVID and non-COVID respiratory infections, with a trend for more frequent symptoms and particularly cognitive symptoms following COVID.

